# Revisiting superiority and stability metrics of cultivar performances using genomic data: derivations of new estimators

**DOI:** 10.1186/s13007-024-01207-1

**Published:** 2024-06-06

**Authors:** Humberto Fanelli Carvalho, Simon Rio, Julian García-Abadillo, Julio Isidro y Sánchez

**Affiliations:** 1 Centro de Biotecnología y Genómica de Plantas (CBGP, UPM-INIA)-Universidad Politécnica de Madrid (UPM)-Instituto Nacional de Investigación y Tecnologia Agraria y Alimentaria (INIA), Campus de Montegancedo-UPM, 28223 Pozuelo de Alarcón, Madrid Spain; 2 CIRAD, UMR AGAP Institut, 34398 Montpellier, France; 3 UMR AGAP Institut, Univ Montpellier, CIRAD, INRAE, Institut Agro, Montpellier, France

**Keywords:** Genotype-by-environment, Genomic prediction, Environmental variance, Ecovalence, Finlay–Wilkinson regression coefficient, Lin–Binns superiority measure

## Abstract

The selection of highly productive genotypes with stable performance across environments is a major challenge of plant breeding programs due to genotype-by-environment (GE) interactions. Over the years, different metrics have been proposed that aim at characterizing the superiority and/or stability of genotype performance across environments. However, these metrics are traditionally estimated using phenotypic values only and are not well suited to an unbalanced design in which genotypes are not observed in all environments. The objective of this research was to propose and evaluate new estimators of the following GE metrics: *Ecovalence*, *Environmental Variance*, *Finlay–Wilkinson* regression coefficient, and *Lin–Binns* superiority measure. Drawing from a multi-environment genomic prediction model, we derived the best linear unbiased prediction for each GE metric. These derivations included both a squared expectation and a variance term. To assess the effectiveness of our new estimators, we conducted simulations that varied in traits and environment parameters. In our results, new estimators consistently outperformed traditional phenotype-based estimators in terms of accuracy. By incorporating a variance term into our new estimators, in addition to the squared expectation term, we were able to improve the precision of our estimates, particularly for *Ecovalence* in situations where heritability was low and/or sparseness was high. All methods are implemented in a new R-package: *GEmetrics*. These genomic-based estimators enable estimating GE metrics in unbalanced designs and predicting GE metrics for new genotypes, which should help improve the selection efficiency of high-performance and stable genotypes across environments.

**Supplementary Information:**

The online version contains supplementary material available at 10.1186/s13007-024-01207-1.

## Background

The performance of cultivars is dependent on selective breeding or genetic modification of a specific genotype to display distinct agronomic traits. Such performance varies, given different locations and management conditions (e.g. temperature, rainfall, or nitrogen content), a phenomenon referred to as genotype-by-environment (GE) interaction [1]. As this interaction complicates the selection of suitable genotypes for quantitative traits, it delays the cultivation and distribution of new crop varieties [2]. A good characterization of GE interactions is fundamental to a better understanding of the relationship between genetic and environmental factors that shape complex crop traits [3]. Multi-environment trials (MET), or experimental sets that evaluate a population of genotypes in various conditions, help characterize GE interactions and facilitate the decision-making process of breeders [4, 5].

Several metrics have been proposed over the years to characterize the stability of genotypes across multiple environments. Two concepts of stability have been established: static stability, in which a stable genotype maintains a constant performance regardless of the effect of the environment, and dynamic stability, in which the genotype follows environmental response with constant deviation [6]. Accordingly, we can categorize GE metrics as static, such as *Environmental Variance* defined as the variance in performance of a genotype across environments [7], or dynamic such as *Ecovalence* defined as the contribution of a genotype to the GE sum of squares of an analysis of variance (ANOVA) [8]. Additionally, the *Finlay–Wilkinson* regression coefficient of the performance of a genotype on environmental means can be used to assess stability according to the two concepts: static around zero and dynamic around one [9]. A cultivar’s potential is assessed not only by the stability of its performance across environments but also by its superiority to other cultivars. Although mean cultivar performance remains the gold standard in breeding, other measures have been proposed, such as *Lin–Binns* superiority measure [10]. In the latter, cultivars that perform particularly poorly in one or more environments are penalized in comparison to those with average performance. Stability and superiority measures are traditionally estimated using phenotypic observations only. In practice, the estimates of GE combinations are obtained from a linear model with fixed genotyped and environment effects [6, 11].

Linear mixed models have been employed to estimate breeding values (BVs) considering genotype effects as random [12, 13]. The model’s advantages include shrinkage towards the mean when the reliability of the BV estimates are low, the capability to handle unbalanced data, and the ability to estimate genetic and non-genetic components of variance [14]. They have proven their worth across diverse datasets, estimating multi-generation indices across years and environments [15], dealing with highly unbalanced experimental design as p-rep [16], and estimating quantitative genetics model parameters using Bayesian inference [17].

Using dense molecular marker panels, genomic prediction has been suggested to predict the breeding value of unobserved genotypes, which revolutionized selection methods for quantitative traits [18]. The incorporation of a genomic relationship kernel into mixed models has amplified genomic selection efficiency [19]. In the context of METs, GE interactions can be viewed as resulting from the differential expression of quantitative trait loci (QTL) in relation to environmental variables. The mixed model’s flexibility with genomic data aids in predicting GE effects and unobserved genotype performance in different environments [20–24].

In the prevailing genomic era, utilizing only adjusted means from a standard linear model to estimate traditional GE metrics does not fully exploit the extensive information available. For instance, [25] proposed a Bayesian approach to estimate the Finlay and Wilkinson regression coefficient while taking genomic information into account. Alternatively, one approach to refine GE metric estimates is to use environment-specific GEBVs (env-GEBVs, i.e. genomic BLUPs of environment-specific BVs) instead of merely relying on adjusted means. Within the linear mixed model context, the expectation of a random term, given observed data, equates to its BLUP [13, 26]. From this definition, we can compute a genomic BLUP for GE metrics that encompasses both a squared expectation component and a variance component. As far as we are aware, no previous work has presented or evaluated these new GE metric estimators.

In this study, we (i) derived novel estimators for GE stability and superiority metrics, comparing their efficacy in approximating the *true* GE metric values - those theoretically derived from environment-specific breeding values (env-BVs)—against traditional phenotype-based estimators; (ii) assessed how various traits and environmental parameters influence the performance of these estimators; and (iii) determined the predictability of GE stability and superiority metrics for genotypes not observed in any of the environments. Our analyses utilize three public empirical datasets-maize, oat, and sorghum-and are further supported by simulations.

## Methods

### Derivation of GE metric estimators

In this study, we focused on five GE metrics used to assess the stability and superiority of genotype performances across environments. To develop new estimators, our approach consisted of (i) presenting formulas to calculate *true* GE metrics, i.e. the values that one would obtain by calculating them directly using env-BVs, (ii) proposing a genomic prediction model adapted to METs data, and (iii) calculate the expectation of GE metrics conditional on the phenotypes based on the genomic prediction model, i.e. BLUPs of the GE metrics. According to this approach, GE metrics are defined as random variables. Note that they could also be defined as fixed or variance model parameters [27–29]. The advantage of treating GE metrics as random variables is that it eliminates the need to fit a model that specifically accounts for stability parameters. Instead, GE metric estimates can be obtained from any multi-environment genomic prediction model that considers GE interactions.

#### GE metrics

Let us assume a METs dataset with *N* genotypes evaluated in *J* environments. Each genotype *i* has an env-BV $G_{ij}$ specific to each environment *j*, as a result of environmental effects and GE interactions. In what follows, the average over *N* genotypes or over *J* environments is indicated by a dot in the BV subscripts, e.g. the BV corresponds to the average env-BV of a genotype *i* over environments is referred to as $G_{i.}=\frac{1}{J}\sum ^J_{j=1}G_{ij}$, and the average BV over all genotypes is referred to as $G_{..}=\frac{1}{JN}\sum ^N_{i=1}\sum ^J_{j=1}G_{ij}$.

The first GE metric $L_{i}$ considered in our study is the *Lin–Binns* superiority measure [10]:

1 $$\begin{aligned} L_{i}=\dfrac{1}{2J}\sum ^J_{j=1}\left( G_{ij}-G_{r_{j}j}\right) ^2 \end{aligned}$$

which aims, for each genotype *i*, to average over all environments the squared difference between its env-BV and that of the best-performing/reference genotype $r_{j}$ in environment *j*. Compared to a simple average of genotype performances over environments, it tends to penalize genotypes that perform particularly poorly in given environments.

The second GE metric $S_i$ is a measure of static stability named *Environmental Variance* [7]:

2 $$\begin{aligned} S_{i}=\dfrac{1}{J-1}\sum ^J_{j=1}\left( G_{ij}-G_{i.}\right) ^2 \end{aligned}$$

which aims to calculate the variance of the performance of each genotype *i* over environments.

The third GE metric $W_i$ is a measure of dynamic stability named *Ecovalence* [8]:

3 $$\begin{aligned} W_{i}=\sum ^J_{j=1}\left( G_{ij}-G_{i.}-G_{.j}+G_{..}\right) ^2 \end{aligned}$$

which aims to quantify the contribution of a genotype to the GE sum of squares of an ANOVA, i.e. to which extent env-BVs deviate non-uniformly from the mean of each environment.

The last GE metric $B_{i}$ presented in our study is the *Finlay–Wilkinson* regression coefficient [9]:

4 $$\begin{aligned} B_{i}=\dfrac{\sum ^J_{j=1}\left( G_{ij}-G_{i.}\right) \left( G_{.j}-G_{..}\right) }{\sum ^J_{j=1}\left( G_{.j}-G_{..}\right) ^2} \end{aligned}$$

which aims, for each genotype *i*, to regress env-BVs on the environment means.

#### Genomic prediction model

We decomposed each env-BV $G_{ij}$ into an environment mean parameter $\mu _j$ and a centered env-BV $U_{ij}$:

5 $$\begin{aligned} G_{ij}=\mu _j+U_{ij} \end{aligned}$$

We then modeled the phenotypes as [30]:

6 $$\begin{aligned} \varvec{y=X\beta +Zu+e} \end{aligned}$$

where $\varvec{y}^T=\left( \varvec{y}_1^T,...,\varvec{y}_J^T\right)$ is the concatenated vector of phenotypes in *J* environments with *P* plots each, $\varvec{\beta }=(\mu _1,...,\mu _J)^T$ is the vector of fixed environment means, $\varvec{X}$ is the design matrix for fixed effects, $\varvec{u}^T=\left( \varvec{u}_1^T,...,\varvec{u}_J^T\right)$ is the concatenated vector of random centered env-BVs with $\varvec{u}\sim \mathscr {N}(0,\varvec{\Sigma }_{G})$ and $\varvec{\Sigma }_{G}$ being the covariance matrix of $\varvec{u}$, $\varvec{Z}$ is the incidence matrix linking phenotypic observations to environment-specific breeding values, $\varvec{e}$ is the vector of errors with $\varvec{e}\sim \mathscr {N}(0,\varvec{\Sigma }_{E})$ and $\varvec{\Sigma }_{E}$ being the covariance matrix of $\varvec{e}$. Independence is assumed between $\varvec{u}$ and $\varvec{e}$, and the covariance matrix between phenotypic observations is: $\varvec{\Sigma }_{Y}=\varvec{Z}\varvec{\Sigma }_{G}\varvec{Z}^T+\varvec{\Sigma }_{E}.$

Let us assume $\varvec{\Sigma }_{G}=\varvec{\Omega }_{G}\bigotimes \varvec{A}$ where $\varvec{A}$ is the genomic relationship matrix between genotypes (or any genetic relationship matrix between them), and $\varvec{\Omega }_{G}$ is the genetic covariance matrix between environments used to account for GE interactions. Similarly, let us assume $\varvec{\Sigma }_{E}=\varvec{\Omega }_{E}\bigotimes \varvec{I}_P$ where $\varvec{I}_P$ is the identity matrix of dimension *P* and $\varvec{\Omega }_{E}$ is the error covariance matrix between environments. Note that $\varvec{\Omega }_{E}$ can be written using a Kronecker product as the number of plots/observations *P* is considered constant in all environments. We will assume the following form for $\varvec{\Omega }_{G}$ and $\varvec{\Omega }_{E}$:

$$\bullet \quad \varvec{\Omega }_{G}=\begin{bmatrix}\sigma _{G_{1}}^2 &{} .. &{}\sigma _{G_{1,J}}\\ .. &{} .. &{} .. \\ \sigma _{G_{J,1}} &{} .. &{} \sigma _{G_{J}}^2\end{bmatrix}$$

$$\bullet \quad \varvec{\Omega }_{E}=\begin{bmatrix}\sigma _{E_{1}}^2 &{} .. &{}0\\ .. &{} .. &{} .. \\ 0 &{} .. &{} \sigma _{E_{J}}^2\end{bmatrix}$$

where $\sigma _{G_{j}}^2$ is the genetic variance in environment *j*, $\sigma _{G_{j,j'}}$ is the genetic covariance between environments *j* and $j'$, and $\sigma _{E_{j}}^2$ is the error variance in environment *j*. Let us also define $\rho _{j,j'}=\dfrac{\sigma _{G_{j,j'}}}{\sigma _{G_{j}}\sigma _{G_{j'}}}$ as the genetic correlation between environments *j* and $j'$.

#### BLUP of GE metrics

Let us first consider the env-GEBVs (i.e. the BLUPs of env-BVs), which are the cornerstone of MET genomic prediction. They can be obtained by considering the distribution of $G_{ij}$ conditional on $\varvec{y}$, which is Gaussian with expectation:

7 $$\begin{aligned} \textrm{E}\left( G_{ij}|\varvec{y}\right) =\mu _j+\left[ \varvec{\Sigma }_G\varvec{Z}^T\varvec{\Sigma }_Y^{-1} \left( \varvec{y}-\varvec{X}\varvec{\beta }\right) \right] _{ij} \end{aligned}$$

and variance:

8 $$\begin{aligned} \textrm{Var}\left( G_{ij}|\varvec{y}\right) =\left[ \varvec{\Sigma }_G-\varvec{\Sigma }_G\varvec{Z}^T \varvec{\Sigma }_Y^{-1}\varvec{Z}\varvec{\Sigma }_G\right] _{ij,ij}=\left[ \varvec{P}\right] _{ij,ij}=P_{ij,ij} \end{aligned}$$

In practice, $\varvec{\beta }$ is unknown and is thus replaced by its best linear unbiased estimate (BLUE):

9 $$\begin{aligned} \widehat{\varvec{\beta }}=\left( \varvec{X}^{T}\varvec{\Sigma }_Y^{-1}\varvec{X}\right) ^{-1}\varvec{X}^{T}\varvec{\Sigma }_Y^{-1}\varvec{y} \end{aligned}$$

which, after replacing fixed effects by their estimates, gives the following formula for env-GEBVs:

10 $$\begin{aligned} \widehat{G}_{ij}=\widehat{\mu }_j+\left[ \varvec{\Sigma }_{G}\varvec{Z}^T\varvec{M}\varvec{y}\right] _{ij} \end{aligned}$$

with:

11 $$\begin{aligned} \varvec{M}=\varvec{\Sigma }_Y^{-1}-\varvec{\Sigma }_Y^{-1}\varvec{X}\left( \varvec{X}^T\varvec{\Sigma }_Y^{-1}\varvec{X}\right) ^{-1}\varvec{X}^T\varvec{\Sigma }_Y^{-1} \end{aligned}$$

Note that $P_{ij,ij}$ in Eq. (8) can be seen as the prediction error variance (PEV) associated with the prediction of $G_{ij}$ for which $\varvec{\beta }$ would be known [i.e. not estimated using Eq. (9)].

The BLUP of the average env-BVs (*Average*) over environments can be calculated as:

12 $$\begin{aligned} \textrm{E}\left( G_{i.}|\varvec{y}\right) =\frac{1}{J}\sum ^J_{j=1}\widehat{G}_{ij} \end{aligned}$$

Let us now consider a squared linear combination of env-BVs named $Q^2$. One can calculate the BLUP of $Q^2$ and split it into two terms following the König–Huygens theorem:

13 $$\begin{aligned} {\textrm{E}\left( Q^2|\varvec{y}\right) =\left[ \textrm{E}\left( Q|\varvec{y}\right) \right] ^2 + \textrm{Var}\left( Q|\varvec{y}\right) } \end{aligned}$$

with a first term corresponding to the squared expectation of *Q* conditional on phenotypes (i.e. the squared BLUP of *Q*) and a second term corresponding to the variance of *Q* conditional on phenotypes. This result can be used to calculate the BLUP of each GE metric, which involves quantities previously presented (see matrix derivations in Appendix and algebraic derivations in Supplementary File S1):

14 $$\begin{aligned} \textrm{E}\left( L_i|\varvec{y}\right) =\dfrac{1}{2J}\sum ^J_{j=1}\left( \widehat{G}_{ij} -\widehat{G}_{r_{j}j}\right) ^2+\dfrac{1}{2J}\sum ^J_{j=1}\left( P_{ij,ij} + P_{r_{j}j,r_{j}j} - 2P_{ij,r_{j}j}\right) \end{aligned}$$

and

15 $$\begin{aligned} \textrm{E}\left( S_{i}|\varvec{y}\right) =\dfrac{1}{J-1}\sum ^J_{j=1}\left( \widehat{G}_{ij} -\widehat{G}_{i.}\right) ^2+\dfrac{1}{J-1}\sum ^J_{j=1}\left( P_{ij,ij}-\dfrac{1}{J}\sum ^J_{j'=1}P_{ij,ij'}\right) \end{aligned}$$

and

16 $$\begin{aligned} \textrm{E}\left( W_{i}|\varvec{y}\right) & =\sum ^J_{j=1}\left( \widehat{G}_{ij} -\widehat{G}_{i.}-\widehat{G}_{.j}+\widehat{G}_{..}\right) ^2 \\& \quad + \sum ^J_{j=1}\left[ P_{ij,ij} - \frac{1}{J}\sum ^J_{j'=1}P_{ij,ij'} - \sum ^N_{i'=1}\left( \frac{2}{N}P_{ij,i'j} -\frac{1}{N^2}\sum ^N_{i''=1}P_{i'j,i''j}\right) \right. \\&\quad \left. + \frac{1}{J}\sum ^J_{j'=1}\sum ^N_{i'=1}\left( \frac{2}{N}P_{ij,i'j'} -\frac{1}{N^2}\sum ^N_{i''=1}P_{i'j,i''j'}\right) \right] \end{aligned}$$

and

17 $$\begin{aligned} \textrm{E}\left( B_{i}|\varvec{y}\right) \approx \dfrac{\sum ^J_{j=1}\left( \widehat{G}_{ij} -\widehat{G}_{i.}\right) \left( \widehat{G}_{.j}-\widehat{G}_{..}\right) +\sum ^J_{j=1} \left( \dfrac{1}{N}\sum ^N_{i'=1}P_{ij,i'j}-\dfrac{1}{JN}\sum ^J_{j'=1}\sum ^N_{i'=1}P_{ij,i'j'} \right) }{\sum ^J_{j=1}\left( \widehat{G}_{.j}-\widehat{G}_{..}\right) ^2+\sum ^J_{j=1} \left( \dfrac{1}{N^2}\sum ^N_{i'=1}\sum ^N_{i''=1}P_{i'j,i''j} -\dfrac{1}{JN^2}\sum ^J_{j'=1}\sum ^N_{i'=1}\sum ^N_{i''=1}P_{i'j,i''j'}\right) } \end{aligned}$$

For the BLUP of the first three GE metrics, the first part of their expression corresponds to the squared expectation term and consists of replacing env-BVs in Eq. (1), Eqs. (2) and (3) by env-GEBVs. The rest of the expression corresponds to the variance term and involves summing elements of the $\varvec{P}$ matrix from Eq. (8). For the BLUP of *Finlay–Wilkinson*, which consists of a ratio between two terms, a simplifying assumption was made that the expectation of this ratio was approximately equal to the ratio of the expectation of the two terms. Each term of the ratio was then considered similarly to the other GE metrics. Note that this approximation corresponds to the first order Taylor series approximation of the BLUP of *Finlay–Wilkinson* [31].

In this study, we evaluated three estimators for each GE metric. First, we considered the traditional “No-Geno” estimator, which consists of replacing $G_{ij}$ by estimates based solely on phenotypic information, i.e. BLUEs of genotypic effects, in Eqs. 1–4. Based on our theoretical results, we then proposed two novel estimators accounting for genomic information: “Geno-Exp” which incorporates only the squared expectation term of Eqs. 14–17, and “Geno-Exp-Var” which encompasses all quantities from the above-mentioned formulas. Note that only the “Geno-Exp-Var” estimators are unbiased in the sense that the expectation (on the phenotypes) of the BLUPs of GE metrics equals the expectation of the GE metrics [32], e.g. $\textrm{E}_{\varvec{y}}\left[ \textrm{E}\left( L_{i}|\varvec{y}\right) \right] =\textrm{E}\left( L_{i}\right)$.

### Materials

To validate the new GE estimators, we utilized both simulated and empirical datasets. We performed simulations based on the model in Eq. (6), varying the following parameters: (i) the standard normal deviation of the environment means ($\sigma _{\mu }$), where $\mu _{j} \sim \mathscr {N}(100, \sigma ^2_{\mu })$ independent and identically distributed (IID), (ii) the genetic correlation between 10 environments pairwise ($\rho _{j,j'}$), (iii) the heritability in each environment ($h^2_j$), where $h_{j}^2 = c + d_j$ with a constant *c* and a deviation $d_j\sim \mathscr {U}(-0.2, 0.2)\hspace{2pt}$ IID, and (iv) the sparsness of the dataset defined as the percentage of missing genotype-environment combinations. In this study, to examine the influence of the simulation parameters, we first considered a basic scenario with intermediate parameter levels: $\sigma _{\mu }$ = 1, $\rho _{j,j'}$ = 0.5 for all $j\ne j'$, $h_{j}^2 = 0.5 + d_j$, and sparseness = 0%. We studied the influence of each parameter by modulating them one by one, considering the following levels: $\sigma _{\mu } \in \{0.1,1,10\}$, $\rho _{j,j'} \in \{0.2,0.5,0.8\}$ for all $j\ne j'$, $c \in \{0.2,0.5,0.8\}$ for $h_{j}^2$, and sparseness $\in \{0\%,25\%,50\%,75\%\}$ (Supplementary Table S1).

For simulated traits, the vector of random centered env-BVs was generated using the product between the Cholesky decomposition of $\varvec{\Sigma }_{G}$ (scaled with $\sigma ^2_{G_{j}}=1$ for all *j* and $\rho _{j,j'}$ chosen according to the simulation scenario) and a vector of independent draws from a standard normal distribution [30]. As genomic data, we used data from a 200-genotyped wheat population, which consisted of 1279 SNPs obtained through diversity array technology (DArT) sequencing [33].

Three publicly available datasets were used to validate the reliability of our novel GE metric estimators, as presented in Table 1. These datasets span various species, genotypes, and environments, all genotyped-by-sequencing. They vary in the number of genotypes (111 to 699), environments (3 to 16), and molecular markers (17,288 to 58,960). For each dataset, yield and plant height traits were evaluated in randomized complete block designs in each environment. Adjusted means were calculated to correct for within-environment spatial/design effects and were used directly as response variables in the genomic prediction model. Further details on the experiments and adjusted means are available in Table 1.

**Table 1:** Summary of the datasets utilized in this study

Dataset	Genotypes	Markers	Environments	References
Maize	133/111^a^	22,432	15/16^b^	[34], G2F:GE (2014-2017)
Oat	699	17,288	3	[35]
Sorghum	133	58,960	3	[36]

“Dataset” refers to the species included in each dataset, “Genotypes” represent the number of genotypes, “Markers” indicate the number of markers, “Environments” indicates the number of environments, and “Reference” refers to the published study associated with each dataset. In each dataset, the population was evaluated for yield and plant height

^a^133 genotypes were evaluated for yield and 111 for plant height

^b^From the 71 available environments, 15 were selected for yield and 16 for plant height, all based on a randomized complete block design (RCBD). This selection was made to minimize the number of missing values

#### Workflow scheme

**Fig. 1:**
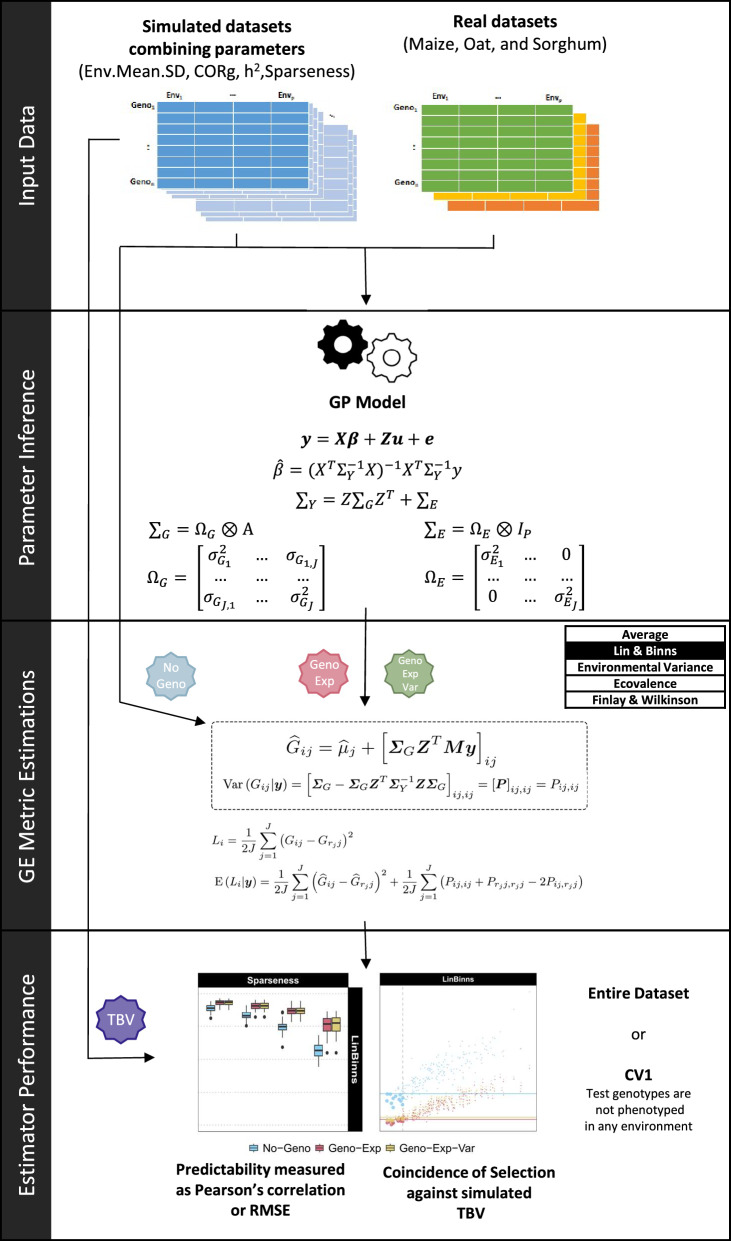
Diagram illustrating the workflow steps. *Input Data:* either simulated datasets considering a combination of trait and environment parameters ($\sigma _\mu$, $\rho _{j,j'}$, $h_{j}^2$ and sparseness), or empirical datasets (maize, oat, and sorghum). *Parameter Inference:* obtain estimates for fixed parameters ($\varvec{\beta }$), and variance component ($\varvec{\Omega }_{G}$ and $\varvec{\Omega }_{E}$), used to calculate $\varvec{\Sigma }_{G}$, $\varvec{\Sigma }_{E}$, and $\varvec{\Sigma }_{Y}$. *Genotype-by-Environment (GE) Metric Estimations:* calculation of env-GEBVs and conditional variance of env-BVs, which are used to estimate the following GE metrics: *Lin–Binns*, *Environmental Variance*, *Ecovalence*, *Finlay–Wilkinson*, and *Average*. GE metrics were estimated using: an estimator based on phenotypic information only (No-Geno), an estimator accounting for genomic information through env-GEBVs (Geno-Exp), and a last estimator accounting for genomic information through env-GEBVs and conditional variance of env-BVs (Geno-Exp-Var). *GE Estimator Performance:* comparing the performance of GE metric estimators using the correlation, the root-mean-square error (RMSE), and the selection coincidence between GE metric estimates and GE metric reference values

In this study, the workflow was divided into four stages: input data, parameter inference, GE metrics estimation, and estimator performance (Fig. 1). The specific details are expounded upon as follows:

- Input data: Three empirical datasets were considered (Table 1), as well as simulated datasets considering combinations of trait and environment parameter levels: $\sigma _\mu$, $\rho _{j,j'}$, $h_{j}^2$, and sparseness (Supplementary Table S1). A detailed description of the input datasets and the simulation procedure is presented in the previous section.
- Parameter inference: The second phase involved adjusting the model from Eq. (6) on the input data. From this model, estimates were obtained for fixed effects $\varvec{\beta }$ and for the $\varvec{\Omega }_{G}$ and $\varvec{\Omega }_{E}$ variance components, which are used to calculate $\varvec{\Sigma }_{G}$, $\varvec{\Sigma }_{E}$, and $\varvec{\Sigma }_{Y}$. A Bayesian multivariate approach based on a Gibbs sampler was used to infer parameters [37].
- GE metrics estimations: The third step consisted of estimating the GE metrics. New estimators require the calculation of env-GEBVs (Eq. 10) as well as the conditional variance of env-BVs (Eq. 8). Two genomic-based estimators were derived: one based only on the squared expectation term (Geno-Exp) and a second also involving the variance term (Geno-Exp-Var). Note that the phenotype-based estimator (No-Geno) only requires phenotypic data. For the latter, missing genotype-environment combinations were imputed with the average value of the environment, unlike genomic-based estimators for which they could always be predicted.
- Estimator performance: The fourth step entailed comparing the different estimators (No-Geno, Geno-Exp, and Geno-Exp-Var) in terms of the precision of their estimates. Their performance was evaluated based on the correlation and the root-mean-squared error (RMSE) between the GE metric estimates and either (i) the *true* GE metrics based on env-BVs for simulated datasets or (ii) the GE-metrics calculated based on adjusted means (i.e. No-Geno estimates) for empirical datasets. Therefore, the No-Geno estimators were not evaluated for empirical datasets, as these estimates were used as reference values for validation. Using simulation, a selection coincidence was determined by calculating the proportion of common genotypes obtained with a given selection intensity (e.g. 10% best) between genotypes selected based on GE metric estimates and GE metric *true* values. For the genomic prediction of GE metrics of non-observed genotypes, cross-validation (CV) was performed by repeatedly splitting the datasets into training and test sets, considering 50 repetitions. The CV approach consisted of discarding all phenotypic data associated with test genotypes, commonly referred to as CV1 in MET experiments [20]. Different training set sizes were defined in terms of percentage of the full dataset (25%, 50%, and 75%) and precision criteria were calculated based on the test set only.

#### Statistical software

We conducted all analysis using the R programming language version 4.2.2 [38]. We developed a new R-package named “GEmetrics” available from the CRAN and from GitHub (https://github.com/TheRocinante-lab/GEmetrics), providing functions for simulating data and calculating new GE metric estimators. The inference of model parameters has been performed using the R package “BGLR” [39], with the “Multitrait” function and the following parameters: nIter = 20000, burnIn = 2000, and thin = 2. Data visualization and harmonization were carried out using the *tidyverse* R-package [40]. The plots and figures in this study are colorblind-friendly, following the *Safe* pallet of color from the *colorblindcheck* R-package [41].

## Results

### Simulation-based evaluation of estimator performance

*Simulation of trait and environment parameters:* In this study, to evaluate the influence of trait and environment parameters on the precision of GE metric estimates, we simulated MET data varying the following parameters: standard deviation of the environmental means ($\sigma _\mu$), the genetic correlation between environments ($\rho _{j,j'}$), heritability ($h_{j}^2$), and sparseness of the data. We considered a basic scenario with 0% sparseness and intermediate parameter levels for other parameters ($\sigma _\mu$ = 1, $\rho _{j,j'}$ = 0.5, and $h_{j}^2$ = 0.5). We assessed the impact of each parameter by modulating it while keeping the others fixed (Supplementary Table S1).

**Fig. 2:**
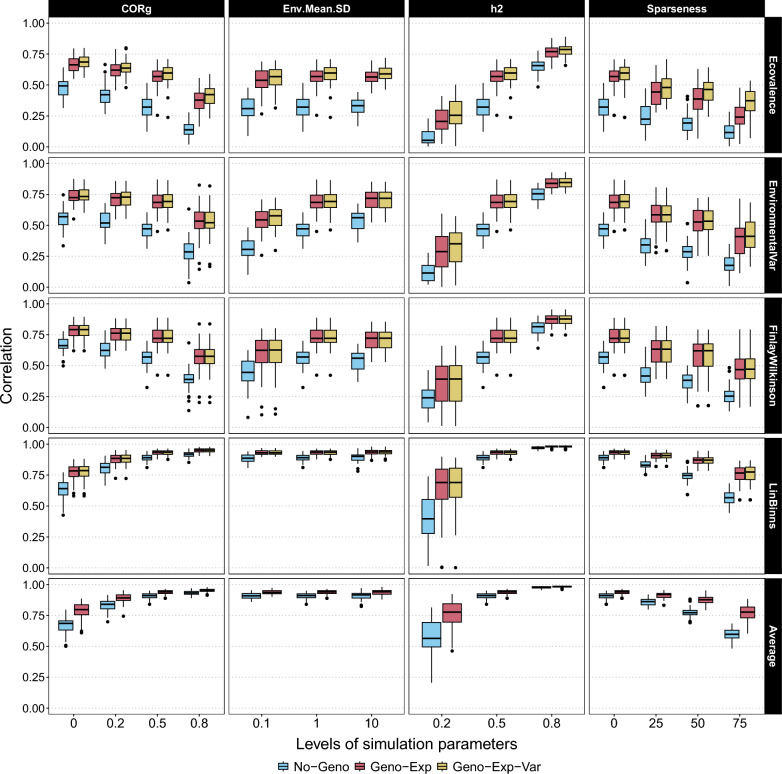
Correlation (y-axis) between the GE metrics calculated from simulated environment-specific breeding values and the estimates obtained from No-Geno, Geno-Exp, and Geno-Exp-Var estimators. Different simulation parameter levels were considered (x-axis): standard deviation of the environmental means ($\sigma _\mu$ = Env.Mean.SD), genetic correlation between environments ($\rho _{j,j'}$ = CORg), heritability ($h_{j}^2$ = h2), and sparseness of the data (0%, 25%, 50%, and 75%). All simulation parameters were modulated one by one around a basic scenario

As a precision criterion, we first used the correlation between GE metric estimates and GE metrics calculated using simulated env-BVs (Fig. 2). Results revealed large differences in precision between GE metrics, regardless of the estimator. The two GE metrics assessing the genotype superiority (i.e. *Lin–Binns* and *Average*) had higher correlations than the other three metrics assessing stability. Increasing the values of $\sigma _\mu$ and $h_{j}^2$ parameters increased the correlations while the sparseness parameter decreased the correlation for all GE metrics. Regarding the genetic correlation $\rho _{j,j'}$, increasing the parameter values was associated with a slightly decreasing correlation for *Ecovalence*, *Environmental Variance*, and *Finlay–Wilkinson* and a slightly increasing correlation for *Lin–Binns* and *Average*. The phenotype-based estimator (No-Geno) underperformed compared with the estimators taking advantage of the genomic information (Geno-Exp and Geno-Exp-Var) for all scenarios. The Geno-Exp-Var estimator had a slightly higher correlation for *Ecovalence* than Geno-Exp for all simulation parameters. This difference between the two estimators was amplified with increasing sparseness and decreasing heritability. Unlike *Ecovalence*, the other GE metrics presented similar correlation values for all simulation scenarios when comparing Geno-Exp and Geno-Exp-Var estimators. We confirmed all these results using the scaled RMSE as an alternative precision criterion (Supplementary Figure S1).

**Fig. 3:**
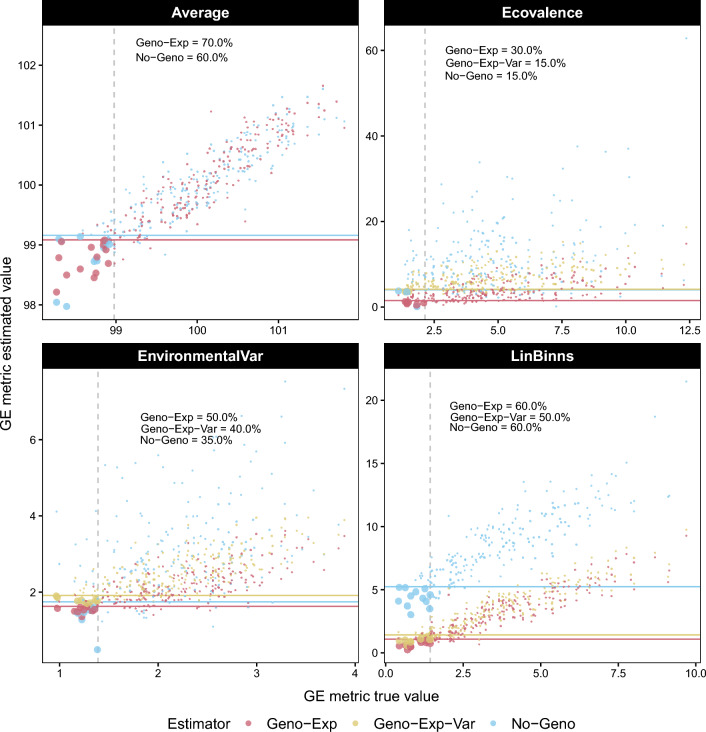
Coincidence of selection for a selection intensity of 10% between genotypes selected using GE metric estimates (y-axis) and with the GE metric values calculated using env-BVs (x-axis). Estimators considered included: No-Geno, Geno-Exp, and Geno-Exp-Var. The threshold indicating the 10% best genotypes is indicated by a grey vertical dashed line for GE metric *true* values and by colored horizontal lines for GE metric estimated. The example shown consists of one simulation run considering the basic scenario with 25% sparseness. The coincidence of selection is indicated in percent for each estimator in the upper left corner of each panel

Coincidence of Selection: To evaluate the ability of the new estimators to identify the most superior and/or stable genotypes among a set of individuals, we defined the selection coincidence as the proportion of common genotypes selected using GE metric estimates and GE metric *true* values, with a given selection intensity. Note that this approach was not applied to *Finlay–Wilkinson*, as stability cannot be assessed in terms of low (or high) values, unlike *Ecovalence*, *Environmental Variance* and *Lin–Binns* for which a low value is desirable. For *Average*, the direction towards which the selection is made depends on the trait. A low value was considered desirable here, but the opposite choice could have been considered without changing the results. The basic simulated scenario was used for comparison, but with 25% sparseness to highlight the differences between Geno-Exp and Geno-Exp-Var, which tend to increase with sparseness. The coincidence of selection was first illustrated based on one simulation run with 10% selection intensity (Fig. 3), displaying coincident genotypes as large bullet points, and further summarized over 50 replicates (Table 2). Overall, the percentages of coincidence of selection presented in Table 2 confirmed the superiority of Geno-Exp and Geno-Exp-Var estimators over No-Geno in all tested scenarios. When compared, Geno-Exp and Geno-Exp-Var estimators generally presented a similar coincidence of selection. However, for *Ecovalence*, Geno-Exp-Var had higher values than Geno-Exp when considering 10%, 15%, and 20% selection intensity (Table 2). Regardless of the estimator, the highest mean values of coincidence were achieved by *Average* followed by *Lin–Binns*, and the lowest values were observed for *Ecovalence*. Similar conclusions could be made considering 0%, 50%, and 75% sparseness (Supplementary Tables S2, S3, and S4).

**Table 2:** Percentage of selection coincidence among genotypes selected based *true* GE metric values (i.e. calculated using env-BVs) or with GE metric estimates obtained using the following estimators: No-Geno, Geno-Exp, and Geno-Exp-Var

Intensity	Estimator	Ecovalence	EnvironmentalVar	LinBinns	Average
5% (10)	No-Geno	7.00% (9.09)	14.20% (10.90)	47.40% (13.67)	54.40% ( 9.07)
	Geno-Exp	15.20% (12.49)	30.40% (17.14)	59.00% (14.18)	63.00% (11.29)
	Geno-Exp-Var	14.60% (13.28)	26.00% (16.54)	60.00% (13.85)	–
10% (20)	No-Geno	12.90% ( 7.57)	22.20% ( 9.85)	58.20% (11.01)	60.70% ( 8.63)
	Geno-Exp	23.20% ( 9.52)	38.60% (13.13)	68.10% (11.29)	71.20% ( 8.42)
	Geno-Exp-Var	23.90% (15.16)	36.90% (14.32)	67.90% (10.88)	–
15% (30)	No-Geno	20.80% ( 6.72)	28.13% ( 9.06)	62.87% ( 9.26)	67.27% ( 6.34)
	Geno-Exp	29.20% ( 9.20)	45.00% (10.11)	74.07% ( 7.54)	73.93% ( 6.86)
	Geno-Exp-Var	31.93% (13.95)	43.07% (10.56)	73.33% ( 7.88)	–
20% (40)	No-Geno	26.80% ( 6.53)	33.25% ( 6.25)	68.10% ( 6.52)	70.20% ( 5.97)
	Geno-Exp	35.15% ( 8.02)	50.10% ( 8.75)	77.10% ( 6.32)	77.30% ( 5.91)
	Geno-Exp-Var	39.75% (10.99)	49.00% ( 9.83)	76.95% ( 6.02)	–

The following selection intensities (Sel. Int.) were considered: 5%, 10%, 15%, and 20%, with the corresponding number of selected genotypes indicated in parentheses. Data was simulated using the basic scenario but with 25% sparseness. The values correspond to averages over 50 replicates, and the standard deviations are shown in parentheses

### Genomic prediction of GE metrics

**Fig. 4:**
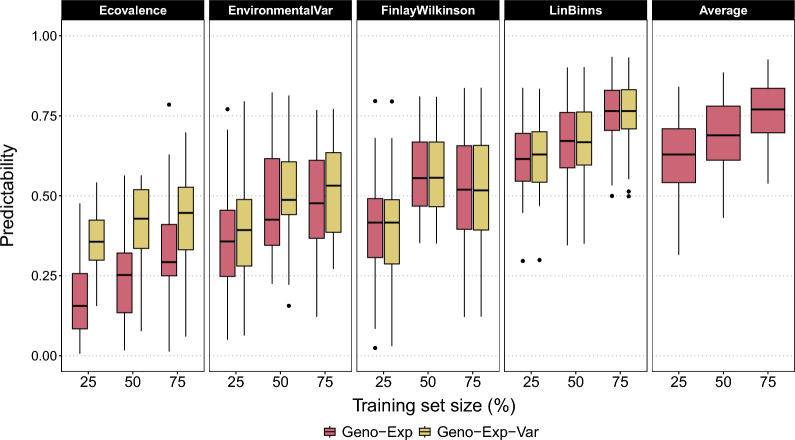
Predictive ability (y-axis) of each GE metric considering the basic simulated scenario. The predictive ability was calculated using the correlation between GE metrics predictions and reference values (i.e. calculated using adjusted means), and was assessed by cross-validation considering different training set sizes (x-axis) in percentage of the total number of genotypes (25%, 50%, and 75%). Two estimators were compared: Geno-Exp and Geno-Exp-Var

Simulation: Using simulations, we investigated the ability of the new estimators Geno-Exp and Geno-Exp-Var to predict the GE metrics of genotypes that were never observed over the whole design. To do so, we performed cross-validation with increasing training set size (Fig. 4). The different estimators were evaluated for their predictive ability, i.e. the correlation between the GE metrics predictions and the *true* GE metric values calculated on the test set genotypes. The predictive ability of all GE metrics increased with training set size regardless of the estimator. The highest values were obtained for *Average*, followed by *LinBinns*, and the lowest values were obtained for *Ecovalence*. For *Environmental Variance*, *Finlay–Wilkinson* and *Lin–Binns*, the performance of the new estimators (Geno-Exp and Geno-Exp-Var) was similar across training set sizes. However, for *Ecovalence*, Geno-Exp-Var outperformed Geno-Exp, in particular for a small training test size of 25% of the total number of genotypes. We confirmed all these results using the scaled RMSE as an alternative precision criterion (Supplementary Figure S2).

**Fig. 5:**
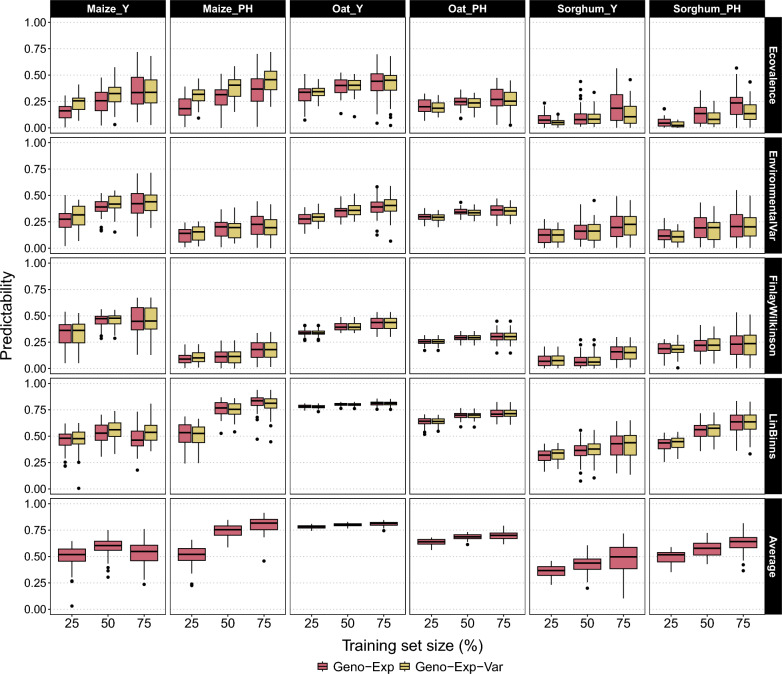
Predictive ability (y-axis) of each GE metric for empirical datasets (Maize, Oat, and Sorghum) evaluated for yield (Y) and plant height (PH). The predictive ability was calculated using the correlation between GE metrics predictions and reference values (i.e. calculated using the adjusted means), and was assessed by cross-validation considering different training set sizes (x-axis) in the percentage of the total number of genotypes (25%, 50%, and 75%). Two estimators were compared: Geno-Exp and Geno-Exp-Var

Empirical Datasets: For all empirical datasets, the adjusted means for yield and plant height were plotted in a scaled boxplot to observe the variability between environments (Supplementary Figure S3). In general, the variability between environments was higher for yield than plant height. Unlike simulations, we did not have access to the *true* value of the GE metrics and could only use the phenotype-based estimates obtained from the No-Geno estimator as a reference. To avoid over-fitting caused by the inclusion of phenotypes in both the training and validation data, we evaluated the performance of the Geno-Exp and Geno-Exp-Var estimators by cross-validation only, accessing the predictive ability (Fig. 5). In general, the predictive abilities obtained were moderate to high for superiority measures (*Lin–Binns* and *Average*) depending on the dataset and the trait. In comparison, the predictive abilities obtained for the other stability GE metrics were low to moderate. Like with simulations, the predictive abilities generally increased with increasing training set size. The performance of Geno-Exp and Geno-Exp-Var was very similar across GE metrics, datasets, and traits. However, for the prediction of *Ecovalence* using the maize dataset, Geno-Exp-Var outperformed Geno-Exp for yield and plant height. The opposite situation was observed for the prediction of *Ecovalence* using the Sorghum for the same two traits. We confirmed all these results using the scaled RMSE as an alternative precision criterion (Supplementary Figure S4).

## Discussion

### New genomics-based GE metric estimators

In this study, we presented new estimators of the following GE metrics: *Environmental Variance* [7], *Ecovalence* [8], *Finlay–Wilkinson* regression coefficient [9], *Lin–Binns* superiority measure [10], and the *Average* performance. Our strategy consisted of calculating the BLUP of each GE metric, defined as random variables, based on any multi-environment genomic prediction model [30]. This approach contrasts with modeling GE metrics as model parameters [27–29], which requires fitting dedicated models.

Except for *Average*, all BLUPs of GE metrics involved a squared expectation and a variance term. From these theoretical results, we have built two types of estimators: Geno-Exp involving only the squared expectation term and Geno-Exp-Var involving both terms. In practice, applying Geno-Exp essentially involves calculating GE metrics using env-GEBVs. These env-GEBVs are common quantities from the application of a multi-environment genomic prediction model. Regarding the variance term, it requires the $\varvec{P}$ matrix from Eq. (8), which can be easily calculated from the model parameter estimates and design and covariance matrices.

The precision of new genomics-based estimators was compared to that of phenotype-based estimators using simulation. Their superiority in terms of estimation accuracy was confirmed for all GE metrics (Fig. 2 and Table 2). The substantial gains observed could probably be explained by the ability of the new genomics-based estimators to borrow information from relatives. Similarly, when focusing on estimating TBVs rather than GE metrics, gains in precision have been observed when combining genomic and phenotypic data [42].

The most interesting feature of our novel estimators is their ability to handle missing values, which is referred to here as sparseness. The gains, compared to the phenotype-based estimator (No-Geno), generally increased with sparseness (Fig. 2). This ability to handle missing values could be extended to the extreme case of predicting the GE metric values of genotypes that have never been observed over the design. Note that this application was only possible using genomics-based estimators. We demonstrated that GE metrics could be predicted with moderate accuracies using simulated and empirical data (Figs. 4 and 5). The accuracies obtained from the empirical data were often lower than those obtained from simulations. This could be explained by the fact that, unlike simulations, we did not have access to *true* GE metric values (i.e. values calculated using env-BVs), but only to phenotype-based estimates as reference values. Similarly, assessing the accuracy of genomic prediction on empirical data sets is impacted by the heritability of phenotype-based BV estimates used to validate predictions. This phenotype-based accuracy is commonly divided by the square root of the heritability to obtain an estimate of the *true* accuracy [43]. However, when it comes to the predictive ability of GE metrics, a simple division by the square root of the heritability is not sufficient, and additional research is required to determine the appropriate adjustment.

Regarding the differences between the two genomics-based estimators, we demonstrated that the use of the complete BLUP (Geno-Exp-Var) was beneficial for *Ecovalence* but less so for the other GE metrics. From a theoretical perspective, the additional variance term included in Geno-Exp-Var is used to add variance to environment-genotype combinations that are poorly predicted. Without this term, a genotype without any observation and unrelated to the rest of the population would be considered perfectly stable according to *Ecovalence* (i.e. estimate close to zero), since it would be predicted at the mean value of each environment. Due to the presence of the variance term, this hypothetical genotype would not be considered perfectly stable as its *Ecovalence* would be estimated above 0.

In practice, we recommend applying the complete genomic BLUP of GE metrics, which we define here as Geno-Exp-Var estimators, because they are unbiased. However, replacing phenotypes with env-GEBVs in traditional phenotype-based estimators has also proven to perform well for most GE metrics, which justifies this approach.

For *Finlay–Wilkinson*, the genomics-based estimators were obtained by approximating the expectation of the ratio from in Eq. 4 by the ratio of the expectation of the numerator and denominator, which consists of a first order Taylor series approximation [31]. In the future, genomics-based estimators of *Finlay–Wilkinson* may be improved by considering higher order Taylor series approximations.

All GE metric estimators could theoretically be applied in the absence of genomic information, provided that the model from Eq. (6) is identifiable. To achieve this, the genomic relationship matrix should be replaced by the identity matrix, and certain genotypes should have replicates in certain environments to separate genetic covariance from error covariance. Because there would be no information shared between relatives, the accuracies associated with predicting GE metrics would probably be inferior to those obtained with genomic information.

### Impact of environment and trait parameters on GE metric estimates

The GE metrics considered in this study serve as tools for assessing both genotype stability and superiority across various environments. These metrics are sensitive to many parameters that characterize the trait and environment. Likewise, the ability to estimate these metrics can be influenced by these same trait and environment parameters, possibly differently depending on the estimator. We have, therefore, investigated the impact of such parameters on the estimation accuracy of our new estimators.

The first parameter considered was the environment-specific heritability ($h^2_j$), which quantifies the proportion of the variance that can be attributed to genetics in a given environment. As expected, the estimation accuracy increased with the heritability, regardless of the GE metric and estimator. Phenotypic data with high heritability are less affected by environmental variations, making their estimation more straightforward. This concept is also crucial in genomic prediction, where high heritability has been shown to improve the accuracy of predictions [44].

The second parameter was the level of sparseness monitoring the amount of information available to estimate the GE metrics. As expected, increasing sparseness was associated with decreasing estimation accuracy, regardless of the GE metric and estimator.

The third parameter considered in this study was the genetic correlation between pairs of environments ($\rho _{j,j'}$). This parameter was used to control the level of GE interaction generating differences in genotype ranking between environments, commonly referred to as crossovers and as opposed to the convergence/divergence of env-BVs according to the environment [45]. The impact of genetic correlations between environments differed depending on whether the GE metric characterizes stability or superiority. For superiority GE metrics, i.e. *Average* and *Lin–Binns*, increasing genetic correlations were associated with increased accuracy (Fig. 2). In the absence of GE interaction, the identification of the best-performing genotype becomes easier as all environments are consistent with each other. For stability measures, increasing genetic correlations was associated with decreased accuracy (Fig. 2). In the absence of GE interaction, all genotypes tend to become equally stable, with differences in performance due solely to shared environmental effects. As a consequence, assessing which genotype is the most stable becomes very difficult, regardless of the estimator.

Finally, the last parameter was the variance of the environment means $\sigma _{\mu }^2$, which controls the proportion of overall variance due to inter-environmental variability relative to intra-environmental variability. This parameter only had an impact on the precision of *Finlay–Wilkinson* and *Environmental Variance* (Fig. 2). For *Finlay–Wilkinson*, a higher variance of the environment means directly impacts the variable on which env-BVs are regressed. The greater the variance, the easier it is to adjust the regression and estimate the regression coefficient.

The main drawback of the genomic prediction model in Eq. 6 is the difficulty associated with estimating variance parameters ($\varvec{\Omega }_G$ and $\varvec{\Omega }_E$ ) correctly when the number of environments becomes large [20]. In such cases, factor analysis approaches may be considered to help structure the genetic covariance matrix $\varvec{\Omega }_G$ and reduce the number of parameters to estimate [46, 47]. However, the simplified GE structure resulting from those approaches may limit the ability to identify unstable genotypes according to specific GE patterns not accounted for in the factor analysis model. Alternatively, if many genotypes per environment are connected by pedigree relationships, it may not be necessary to borrow information across environments, as sufficient data from observed relatives in each environment can enable accurate predictions. In this scenario, it would be feasible to apply a simplified model that ignores genetic covariance between environments, using a heterogeneous diagonal $\varvec{\Omega }_G$ matrix.

## Conclusions

Assessing the superiority and/or the stability of genotype performance over a set of target environments is crucial to making the most suitable varieties available to farmers. An accurate evaluation generally involves costly MET experiments where all genotype-environment combinations are observed repeatedly in each environment. In practice, the data resulting from these experiments is often incomplete due to various issues at different stages of the experiment (e.g. germination defects, plant establishment, floral abortion, etc.). Our new estimators offer an elegant way to deal with incomplete datasets as missing genotype-environment combinations can be predicted in the presence of genomic information. Even when the dataset is complete, we have demonstrated that gains in accuracy can be expected by exploiting information shared between relatives.

METs for which genomic information is available for all genotypes are likely to become the norm in breeding companies. We recommend estimating the proposed GE metrics along with the average genotype performance, which is the most common metric characterizing genotype performance. Two genotypes with similar average performance may exhibit contrasting behavior in terms of stability, and an unstable genotype is susceptible to underperforming in given environmental conditions. Such information is of particular interest from late breeding stages to variety registration. The availability of accurate GE metric estimates provided by our new estimators should facilitate the identification of high-performance and stable genotypes in a range of target environments.

### Supplementary Information

Supplementary Material 1.

Supplementary Material 2.

## Data Availability

All the code and data used in this study can be found in the repository: https://github.com/TheRocinante-lab/Publications/GEmetrics.

## Appendix

Note that *Lin–Binns* (Eq. 1), *Environmental Variance* (Eq. 2), *Ecovalence* (Eq. 3), and the numerator/denominator of *Finlay–Wilkinson* (Eq. 4) can be expressed as:

18 $$\begin{aligned} Q_i=a\times \varvec{g}^T\varvec{C}_i^T\varvec{D}_i\varvec{g} \end{aligned}$$

where $Q_i$ is a GE metric with quadratic form, *a* is a coefficient, $\varvec{g}$ is the vector of env-BVs of dimension *NJ*, $\varvec{C}_i$ and $\varvec{D}_i$ are contrast matrices of dimension $J\times NJ$ specific to individual *i*.

The expected value of $Q_i$ conditional on phenotypes is

19 $$\begin{aligned} \textrm{E}\left( Q_i|\varvec{y}\right) =&a\times \left[ \widehat{\varvec{g}}^T\varvec{C}_i^T\varvec{D}_i\widehat{\varvec{g}} + \textrm{Tr}\left( \varvec{C}_i^T\varvec{D}_i\varvec{P}\right) \right] \end{aligned}$$

where $\widehat{\varvec{g}}$ is the vectors of BLUPs of environment-specific values and “$\textrm{Tr}$” stands for the trace of the matrix (i.e. the sum of diagonal elements).

For *Lin–Binns* (Eq. 1), $\varvec{C}_i=\varvec{D}_i$ has the following form:

20 $$\begin{aligned} \left[ \varvec{C}_i\right] _{j,i'j'} = \left\{ \begin{array}{ c l } 1 &{} \quad \text {if } i'=i \text { and } j=j' \\ -1 &{} \quad \text {if } i'=r_j \text { and } j=j' \\ 0 &{} \quad \text {otherwise} \end{array} \right. \end{aligned}$$

For *Environmental Variance* (Eq. 2), $\varvec{C}_i=\varvec{D}_i$ has the following form:

21 $$\begin{aligned} \left[ \varvec{C}_i\right] _{j,i'j'} = \left\{ \begin{array}{ c l } 1-\frac{1}{J} &{} \quad \text {if } i'=i \text { and } j=j' \\ -\frac{1}{J} &{} \quad \text {if } i'= i \text { and } j\ne j' \\ 0 &{} \quad \text {otherwise} \end{array} \right. \end{aligned}$$

For *Ecovalence* (Eq. 3), $\varvec{C}_i=\varvec{D}_i$ has the following form:

22 $$\begin{aligned} \left[ \varvec{C}_i\right] _{j,i'j'} = \left\{ \begin{array}{ c l } \left( 1-\frac{1}{J}\right) \left( 1-\frac{1}{N}\right) &{} \quad \text {if } i'=i \text { and } j=j' \\ -\frac{1}{J}\left( 1-\frac{1}{N}\right) &{} \quad \text {if } i'=i \text { and } j\ne j' \\ -\frac{1}{N}\left( 1-\frac{1}{J}\right) &{} \quad \text {if } i'\ne i \text { and } j=j' \\ \frac{1}{JN} &{} \text {otherwise} \\ \end{array} \right. \end{aligned}$$

For the numerator of *Finlay–Wilkinson* (Eq. 4), $\varvec{C}_i$ has the same form as Eq. (21) and $\varvec{D}_i$ has the following form:

23 $$\begin{aligned} \left[ \varvec{D}_i\right] _{j,i'j'} = \left\{ \begin{array}{ c l } -\frac{1}{N}\left( 1-\frac{1}{J}\right) &{} \quad \text {if } j= j' \\ -\frac{1}{JN} &{} \quad \text {otherwise} \\ \end{array} \right. \end{aligned}$$
