# Correction: Revisiting superiority and stability metrics of cultivar performances using genomic data: derivations of new estimators

**DOI:** 10.1186/s13007-024-01231-1

**Published:** 2024-07-17

**Authors:** Humberto Fanelli Carvalho, Simon Rio, Julian García-Abadillo, Julio Isidro y Sánchez

**Affiliations:** 1 Centro de Biotecnología y Genómica de Plantas (CBGP, UPM-INIA)-Universidad Politécnica de Madrid (UPM)-Instituto Nacional de Investigación y Tecnologia Agraria y Alimentaria (INIA), Campus de Montegancedo-UPM, 28223 Pozuelo de Alarcón, Madrid, Spain; 2 CIRAD, UMR AGAP Institut, 34398 Montpellier, France; 3 UMR AGAP Institut, Univ Montpellier, CIRAD, INRAE, Institut Agro, Montpellier, France

**Correction: Plant Methods (2024) 20:85**

10.1186/s13007-024-01207-1

The wrong Supplementary file was originally published with this article; it has now been replaced with the correct file.

The original article has been corrected.

### Electronic supplementary material

Below is the link to the electronic supplementary material.

Supplementary Material 1

