# Supplementary material for: Revisiting superiority and stability metrics of cultivar performances using genomic data: derivations of new estimators
Source: Plant Methods. 2024 Jun 6;20:85. doi: 10.1186/s13007-024-01207-1 (PMC11155189; doi:10.1186/s13007-024-01207-1)
Supplement: Supplementary file 1 — Supplementary Material 1. [file 13007_2024_1207_MOESM1_ESM.pdf]

File S1: Derivation of new estimators

Based on the multi-environment genomic prediction model presented in Eq. (6) from the main text, BLUPs of the different superiority and stability GE metrics can be obtained. The following properties are used for the derivations:

- $\mathrm{E}\left(a\sum_{i=1}^{N} X_i\right) = a\sum_{i=1}^{N}\mathrm{E}\left(X_i\right)$
- $\mathrm{Var}\left(a\sum_{i=1}^{N} X_i\right) = a^2\sum_{i=1}^{N}\sum_{i'=1}^{N}\mathrm{Cov}\left(X_i, X_{i'}\right)$
- $\mathrm{E}\left(X^2\right) = \mathrm{E}\left(X\right)^2 + \mathrm{Var}\left(X\right)$

Average

The BLUP of *Average* $\widehat{G}_{i.}$ can be calculated as:

$$\begin{aligned}
\widehat{G}_{i.} &= \mathrm{E}\left(G_{i.}|\boldsymbol{y}\right) \\
&= \mathrm{E}\left(\frac{1}{J}\sum_{j=1}^{J} G_{ij}|\boldsymbol{y}\right) \\
&= \frac{1}{J}\sum_{j=1}^{J}\mathrm{E}\left(G_{ij}|\boldsymbol{y}\right) \\
&= \frac{1}{J}\sum_{j=1}^{J}\widehat{G}_{ij}
\end{aligned}$$

where $\widehat{G}_{ij}$ is the BLUP of env-BVs (env-GEBVs) presented in Eq. (10) from the main text.

Lin-Binns

The BLUP of *Lin-Binns* $\widehat{L}_i$ can be calculated as:

$$\begin{aligned}
\widehat{L}_i &= \mathrm{E}\left(L_i|\boldsymbol{y}\right) \\
&= \mathrm{E}\left(\frac{1}{2J}\sum_{j=1}^{J}\left(G_{ij} - G_{r_jj}\right)^2|\boldsymbol{y}\right) \\
&= \frac{1}{2J}\sum_{j=1}^{J}\mathrm{E}\left(\left(G_{ij} - G_{r_jj}\right)^2|\boldsymbol{y}\right) \\
&= \frac{1}{2J}\sum_{j=1}^{J}\mathrm{E}\left(G_{ij} - G_{r_jj}|\boldsymbol{y}\right)^2 + \frac{1}{2J}\sum_{j=1}^{J}\mathrm{Var}\left(G_{ij} - G_{r_jj}|\boldsymbol{y}\right)
\end{aligned}$$

whose first term can be expressed as:

$$\begin{aligned}
\frac{1}{2J}\sum_{j=1}^{J}\mathrm{E}\left(G_{ij} - G_{r_jj}|\boldsymbol{y}\right)^2 &= \frac{1}{2J}\sum_{j=1}^{J}\left(\mathrm{E}\left(G_{ij}|\boldsymbol{y}\right) - \mathrm{E}\left(G_{r_jj}|\boldsymbol{y}\right)\right)^2 \\
&= \frac{1}{2J}\sum_{j=1}^{J}\left(\widehat{G}_{ij} - \widehat{G}_{r_jj}\right)^2
\end{aligned}$$

and whose second term can be expressed as:

$$\begin{aligned}\frac{1}{2J}\sum_{j=1}^{J}\mathrm{Var}\left(G_{ij}-G_{r_jj}|\boldsymbol{y}\right) &= \frac{1}{2J}\sum_{j=1}^{J}\left[\mathrm{Var}\left(G_{ij}|\boldsymbol{y}\right)+\mathrm{Var}\left(G_{r_jj}|\boldsymbol{y}\right)-2\mathrm{Cov}\left(G_{ij},G_{r_jj}|\boldsymbol{y}\right)\right]\\ &= \frac{1}{2J}\sum_{j=1}^{J}\left(P_{ij,ij}+P_{r_jj,r_jj}-2P_{ij,r_jj}\right)\end{aligned}$$

where $P_{ij,ij}$ is variance of env-BVs conditional on phenotypes presented in Eq. (8).

Environmental Variance

The BLUP of *Environmental Variance* $\widehat{S}_i$ can be calculated as:

$$\begin{aligned}\widehat{S}_i &= \mathrm{E}\left(S_i|\boldsymbol{y}\right)\\ &= \mathrm{E}\left(\frac{1}{J-1}\sum_{j=1}^{J}\left(G_{ij}-G_{i.}\right)^2|\boldsymbol{y}\right)\\ &= \frac{1}{J-1}\sum_{j=1}^{J}\mathrm{E}\left(\left(G_{ij}-G_{i.}\right)^2|\boldsymbol{y}\right)\\ &= \frac{1}{J-1}\sum_{j=1}^{J}\mathrm{E}\left(G_{ij}-G_{i.}|\boldsymbol{y}\right)^2+\frac{1}{J-1}\sum_{j=1}^{J}\mathrm{Var}\left(G_{ij}-G_{i.}|\boldsymbol{y}\right)\end{aligned}$$

whose first term can be expressed as:

$$\begin{aligned}\frac{1}{J-1}\sum_{j=1}^{J}\mathrm{E}\left(G_{ij}-G_{i.}|\boldsymbol{y}\right)^2 &= \frac{1}{J-1}\sum_{j=1}^{J}\left(\mathrm{E}\left(G_{ij}|\boldsymbol{y}\right)-\mathrm{E}\left(G_{i.}|\boldsymbol{y}\right)\right)^2\\ &= \frac{1}{J-1}\sum_{j=1}^{J}\left(\widehat{G}_{ij}-\widehat{G}_{i.}\right)^2\end{aligned}$$

and whose second term can be expressed as:

$$\begin{aligned}\frac{1}{J-1}\sum_{j=1}^{J}\mathrm{Var}\left(G_{ij}-G_{i.}|\boldsymbol{y}\right) &= \frac{1}{J-1}\sum_{j=1}^{J}\left[\mathrm{Var}\left(G_{ij}|\boldsymbol{y}\right)+\mathrm{Var}\left(G_{i.}|\boldsymbol{y}\right)-2\mathrm{Cov}\left(G_{ij},G_{i.}|\boldsymbol{y}\right)\right]\\ &= \frac{1}{J-1}\sum_{j=1}^{J}\left[\mathrm{Var}\left(G_{ij}|\boldsymbol{y}\right)+\mathrm{Var}\left(\frac{1}{J}\sum_{j'=1}^{J}G_{ij'}|\boldsymbol{y}\right)-2\mathrm{Cov}\left(G_{ij},\frac{1}{J}\sum_{j'=1}^{J}G_{ij'}|\boldsymbol{y}\right)\right]\\ &= \frac{1}{J-1}\sum_{j=1}^{J}\left[\mathrm{Var}\left(G_{ij}|\boldsymbol{y}\right)+\frac{1}{J^2}\sum_{j'=1}^{J}\sum_{j''=1}^{J}\mathrm{Cov}\left(G_{ij'},G_{ij''}|\boldsymbol{y}\right)-\frac{2}{J}\sum_{j'=1}^{J}\mathrm{Cov}\left(G_{ij},G_{ij'}|\boldsymbol{y}\right)\right]\\ &= \frac{1}{J-1}\sum_{j=1}^{J}\left[P_{ij,ij}+\frac{1}{J^2}\sum_{j'=1}^{J}\sum_{j''=1}^{J}P_{ij',ij''}-2\frac{1}{J}\sum_{j'=1}^{J}P_{ij,ij'}\right]\\ &= \frac{1}{J-1}\left[\sum_{j=1}^{J}P_{ij,ij}+\frac{1}{J}\sum_{j=1}^{J}\sum_{j'=1}^{J}P_{ij,ij'}-2\frac{1}{J}\sum_{j=1}^{J}\sum_{j'=1}^{J}P_{ij,ij'}\right]\\ &= \frac{1}{J-1}\sum_{j=1}^{J}\left[P_{ij,ij}-\frac{1}{J}\sum_{j'=1}^{J}P_{ij,ij'}\right]\end{aligned}$$

Ecovalence

The BLUP of *Ecovalence* $\widehat{W}_i$ can be calculated as:

$$\begin{aligned}
\widehat{W}_i &= \mathrm{E}\left(W_i|\boldsymbol{y}\right) \\
&= \mathrm{E}\left(\sum_{j=1}^{J}\left(G_{ij}-G_{i.}-G_{.j}+G_{..}\right)^2|\boldsymbol{y}\right) \\
&= \sum_{j=1}^{J}\mathrm{E}\left(\left(G_{ij}-G_{i.}-G_{.j}+G_{..}\right)^2|\boldsymbol{y}\right) \\
&= \sum_{j=1}^{J}\mathrm{E}\left(G_{ij}-G_{i.}-G_{.j}+G_{..}|\boldsymbol{y}\right)^2+\sum_{j=1}^{J}\mathrm{Var}\left(G_{ij}-G_{i.}-G_{.j}+G_{..}|\boldsymbol{y}\right)
\end{aligned}$$

whose first term can be expressed as:

$$\begin{aligned}
\sum_{j=1}^{J}\mathrm{E}\left(G_{ij}-G_{i.}-G_{.j}+G_{..}|\boldsymbol{y}\right)^2 &= \sum_{j=1}^{J}\left(\mathrm{E}\left(G_{ij}|\boldsymbol{y}\right)-\mathrm{E}\left(G_{i.}|\boldsymbol{y}\right)-\mathrm{E}\left(G_{.j}|\boldsymbol{y}\right)+\mathrm{E}\left(G_{..}|\boldsymbol{y}\right)\right)^2 \\
&= \sum_{j=1}^{J}\left(\widehat{G}_{ij}-\widehat{G}_{i.}-\widehat{G}_{.j}+\widehat{G}_{..}\right)^2
\end{aligned}$$

with:

$$\widehat{G}_{.j}=\frac{1}{N}\sum_{i=1}^{N}\widehat{G}_{ij}$$

and with:

$$\widehat{G}_{..}=\frac{1}{NJ}\sum_{i=1}^{N}\sum_{j=1}^{J}\widehat{G}_{ij}$$

and whose second term can be expressed as:

$$\begin{aligned}
\sum_{j=1}^{J} \mathrm{Var}\left(G_{ij} - G_{i.} - G_{.j} + G_{..}|\boldsymbol{y}\right) &= \sum_{j=1}^{J} [\mathrm{Var}\left(G_{ij}|\boldsymbol{y}\right) - 2\mathrm{Cov}\left(G_{ij}, G_{i.}|\boldsymbol{y}\right) - 2\mathrm{Cov}\left(G_{ij}, G_{.j}|\boldsymbol{y}\right) + 2\mathrm{Cov}\left(G_{ij}, G_{..}|\boldsymbol{y}\right) \\
&\quad + \mathrm{Var}\left(G_{i.}|\boldsymbol{y}\right) + 2\mathrm{Cov}\left(G_{i.}, G_{.j}|\boldsymbol{y}\right) - 2\mathrm{Cov}\left(G_{i.}, G_{..}|\boldsymbol{y}\right) + \mathrm{Var}\left(G_{.j}|\boldsymbol{y}\right) \\
&\quad - 2\mathrm{Cov}\left(G_{.j}, G_{..}|\boldsymbol{y}\right) + \mathrm{Var}\left(G_{..}|\boldsymbol{y}\right)] \\
&= \sum_{j=1}^{J} \left[P_{ij,ij} - \frac{2}{J}\sum_{j'=1}^{J} P_{ij,ij'} - \frac{2}{N}\sum_{i'=1}^{N} P_{ij,i'j} + \frac{2}{JN}\sum_{j'=1}^{J}\sum_{i'=1}^{N} P_{ij,i'j'} \right. \\
&\quad + \frac{1}{J^2}\sum_{j'=1}^{J}\sum_{j''=1}^{J} P_{ij',ij''} + \frac{2}{JN}\sum_{j'=1}^{J}\sum_{i'=1}^{N} P_{ij',i'j} - \frac{2}{J^2 N}\sum_{j'=1}^{J}\sum_{j''=1}^{J}\sum_{i'=1}^{N} P_{ij',i'j''} \\
&\quad + \frac{1}{N^2}\sum_{i'=1}^{N}\sum_{i''=1}^{N} P_{i'j,i''j} - \frac{2}{JN^2}\sum_{i'=1}^{N}\sum_{i''=1}^{N}\sum_{j'=1}^{J} P_{i'j,i''j'} \\
&\quad \left. + \frac{1}{J^2 N^2}\sum_{j'=1}^{J}\sum_{j''=1}^{J}\sum_{i'=1}^{N}\sum_{i''=1}^{N} P_{i'j',i''j''} \right] \\
&= \sum_{j=1}^{J} P_{ij,ij} - \frac{2}{J}\sum_{j=1}^{J}\sum_{j'=1}^{J} P_{ij,ij'} - \frac{2}{N}\sum_{j=1}^{J}\sum_{i'=1}^{N} P_{ij,i'j} + \frac{2}{JN}\sum_{j=1}^{J}\sum_{j'=1}^{J}\sum_{i'=1}^{N} P_{ij,i'j'} \\
&\quad + \frac{1}{J}\sum_{j=1}^{J}\sum_{j'=1}^{J} P_{ij,ij'} + \frac{2}{JN}\sum_{j=1}^{J}\sum_{j'=1}^{J}\sum_{i'=1}^{N} P_{ij,i'j'} - \frac{2}{JN}\sum_{j=1}^{J}\sum_{j'=1}^{J}\sum_{i'=1}^{N} P_{ij,i'j'} \\
&\quad + \frac{1}{N^2}\sum_{j=1}^{J}\sum_{i'=1}^{N}\sum_{i''=1}^{N} P_{i'j,i''j} - \frac{2}{JN^2}\sum_{j=1}^{J}\sum_{j'=1}^{J}\sum_{i'=1}^{N}\sum_{i''=1}^{N} P_{i'j,i''j'} \\
&\quad \left. + \frac{1}{JN^2}\sum_{j=1}^{J}\sum_{j'=1}^{J}\sum_{i'=1}^{N}\sum_{i''=1}^{N} P_{i'j,i''j'} \right] \\
&= \sum_{j=1}^{J} \left[P_{ij,ij} - \frac{1}{J}\sum_{j'=1}^{J} P_{ij,ij'} - \sum_{i'=1}^{N}\left(\frac{2}{N}P_{ij,i'j} - \frac{1}{N^2}\sum_{i''=1}^{N} P_{i'j,i''j}\right) \right. \\
&\quad \left. + \frac{1}{J}\sum_{j'=1}^{J}\sum_{i'=1}^{N}\left(\frac{2}{N}P_{ij,i'j'} - \frac{1}{N^2}\sum_{i''=1}^{N} P_{i'j,i''j'}\right) \right]
\end{aligned}$$

Finlay-Wilkinson

The BLUP of *Finlay and Wilkinson* $\widehat{B}_i$ can be calculated as:

$$\begin{aligned}
\widehat{B}_i &= \mathrm{E}\left(B_i|\boldsymbol{y}\right) \\
&= \mathrm{E}\left(\frac{\sum_{j=1}^{J}\left(G_{ij}-G_{i.}\right)\left(G_{.j}-G_{..}\right)}{\sum_{j=1}^{J}\left(G_{.j}-G_{..}\right)^2}|\boldsymbol{y}\right) \\
&\approx \frac{\mathrm{E}\left(\sum_{j=1}^{J}\left(G_{ij}-G_{i.}\right)\left(G_{.j}-G_{..}\right)|\boldsymbol{y}\right)}{\mathrm{E}\left(\sum_{j=1}^{J}\left(G_{.j}-G_{..}\right)^2|\boldsymbol{y}\right)} \\
&\approx \frac{\sum_{j=1}^{J}\mathrm{E}\left(\left(G_{ij}-G_{i.}\right)\left(G_{.j}-G_{..}\right)|\boldsymbol{y}\right)}{\sum_{j=1}^{J}\mathrm{E}\left(\left(G_{.j}-G_{..}\right)^2|\boldsymbol{y}\right)} \\
&\approx \frac{\sum_{j=1}^{J}\mathrm{E}\left(G_{ij}-G_{i.}|\boldsymbol{y}\right)\mathrm{E}\left(G_{.j}-G_{..}|\boldsymbol{y}\right)+\sum_{j=1}^{J}\mathrm{Cov}\left(G_{ij}-G_{i.},G_{.j}-G_{..}|\boldsymbol{y}\right)}{\sum_{j=1}^{J}\mathrm{E}\left(G_{.j}-G_{..}|\boldsymbol{y}\right)^2+\sum_{j=1}^{J}\mathrm{Var}\left(G_{.j}-G_{..}|\boldsymbol{y}\right)}
\end{aligned}$$

whose first numerator term can be expressed as:

$$\begin{aligned}
\sum_{j=1}^{J}\mathrm{E}\left(G_{ij}-G_{i.}|\boldsymbol{y}\right)\mathrm{E}\left(G_{.j}-G_{..}|\boldsymbol{y}\right) &= \sum_{j=1}^{J}\left(\mathrm{E}\left(G_{ij}|\boldsymbol{y}\right)-\mathrm{E}\left(G_{i.}|\boldsymbol{y}\right)\right)\left(\mathrm{E}\left(G_{.j}|\boldsymbol{y}\right)-\mathrm{E}\left(G_{..}|\boldsymbol{y}\right)\right) \\
&= \sum_{j=1}^{J}\left(\widehat{G}_{ij}-\widehat{G}_{i.}\right)\left(\widehat{G}_{.j}-\widehat{G}_{..}\right)
\end{aligned}$$

whose first denominator term can be expressed as:

$$\begin{aligned}
\sum_{j=1}^{J}\mathrm{E}\left(G_{.j}-G_{..}|\boldsymbol{y}\right)^2 &= \sum_{j=1}^{J}\left(\mathrm{E}\left(G_{.j}|\boldsymbol{y}\right)-\mathrm{E}\left(G_{..}|\boldsymbol{y}\right)\right)^2 \\
&= \sum_{j=1}^{J}\left(\widehat{G}_{.j}-\widehat{G}_{..}\right)^2
\end{aligned}$$

whose second numerator term can be expressed as:

$$\begin{aligned}
\sum_{j=1}^{J}\mathrm{Cov}\left(G_{ij}-G_{i.},G_{.j}-G_{..}|\boldsymbol{y}\right) &= \sum_{j=1}^{J}\left[\mathrm{Cov}\left(G_{ij},G_{.j}|\boldsymbol{y}\right)-\mathrm{Cov}\left(G_{ij},G_{..}|\boldsymbol{y}\right)-\mathrm{Cov}\left(G_{i.},G_{.j}|\boldsymbol{y}\right)+\mathrm{Cov}\left(G_{i.},G_{..}|\boldsymbol{y}\right)\right] \\
&= \sum_{j=1}^{J}\left[\frac{1}{N}\sum_{i'=1}^{N}P_{ij,i'j}-\frac{1}{JN}\sum_{j'=1}^{J}\sum_{i'=1}^{N}P_{ij,i'j'}-\frac{1}{JN}\sum_{j'=1}^{J}\sum_{i'=1}^{N}P_{ij',i'j}\right. \\
&\qquad \left.+\frac{1}{J^2N}\sum_{j'=1}^{J}\sum_{j''=1}^{J}\sum_{i'=1}^{N}P_{ij',i'j''}\right] \\
&= \frac{1}{N}\sum_{j=1}^{J}\sum_{i'=1}^{N}P_{ij,i'j}-\frac{1}{JN}\sum_{j=1}^{J}\sum_{j'=1}^{J}\sum_{i'=1}^{N}P_{ij,i'j'}-\frac{1}{JN}\sum_{j=1}^{J}\sum_{j'=1}^{J}\sum_{i'=1}^{N}P_{ij',i'j} \\
&\qquad +\frac{1}{JN}\sum_{j=1}^{J}\sum_{j'=1}^{J}\sum_{i'=1}^{N}P_{ij,i'j'} \\
&= \sum_{j=1}^{J}\left(\frac{1}{N}\sum_{i'=1}^{N}P_{ij,i'j}-\frac{1}{JN}\sum_{j'=1}^{J}\sum_{i'=1}^{N}P_{ij,i'j'}\right)
\end{aligned}$$

and whose second denominator term can be expressed as:

$$
\begin{aligned}
\sum_{j=1}^{J} \mathrm{Var}\left(G_{.j} - G_{..}|\boldsymbol{y}\right) = &\sum_{j=1}^{J} \left[\mathrm{Var}\left(G_{.j}|\boldsymbol{y}\right) + \mathrm{Var}\left(G_{..}|\boldsymbol{y}\right) - 2\mathrm{Cov}\left(G_{.j}, G_{..}|\boldsymbol{y}\right)\right] \\
= &\sum_{j=1}^{J} \left[\frac{1}{N^2} \sum_{i'=1}^{N} \sum_{i''=1}^{N} P_{i'j,i''j} + \frac{1}{J^2 N^2} \sum_{j'=1}^{J} \sum_{j''=1}^{J} \sum_{i'=1}^{N} \sum_{i''=1}^{N} P_{i'j',i''j''} \right. \\
&\left. - \frac{2}{N^2 J} \sum_{j'=1}^{J} \sum_{i'=1}^{N} \sum_{i''=1}^{N} P_{i'j,i''j'} \right] \\
= &\frac{1}{N^2} \sum_{j=1}^{J} \sum_{i'=1}^{N} \sum_{i''=1}^{N} P_{i'j,i''j} + \frac{1}{N^2 J} \sum_{j=1}^{J} \sum_{j'=1}^{J} \sum_{i'=1}^{N} \sum_{i''=1}^{N} P_{i'j,i''j'} \\
&- \frac{2}{N^2 J} \sum_{j=1}^{J} \sum_{j'=1}^{J} \sum_{i'=1}^{N} \sum_{i''=1}^{N} P_{i'j,i''j'} \\
= &\sum_{j=1}^{J} \left(\frac{1}{N^2} \sum_{i'=1}^{N} \sum_{i''=1}^{N} P_{i'j,i''j} - \frac{1}{JN^2} \sum_{j'=1}^{J} \sum_{i'=1}^{N} \sum_{i''=1}^{N} P_{i'j,i''j'} \right)
\end{aligned}
$$
