# Supplementary material for: Revisiting superiority and stability metrics of cultivar performances using genomic data: derivations of new estimators
Source: Plant Methods. 2024 Jun 6;20:85. doi: 10.1186/s13007-024-01207-1 (PMC11155189; doi:10.1186/s13007-024-01207-1)
Supplement: Supplementary file 2 — Supplementary Material 2. [file 13007_2024_1207_MOESM2_ESM.pdf]

Supplementary Figures and Tables

Revisiting superiority and stability metrics of cultivar performances using genomic data: derivations of new estimators

Humberto Fanelli Carvalho[1], Simon Rio[2,3], Julian Garcia-Abadillo[1], and Julio Isidro y Sánchez[1]

[1] Centro de Biotecnologia y Genómica de Plantas (CBGP, UPM-INIA) Universidad Politécnica de Madrid (UPM) - Instituto Nacional de Investigación y Tecnologia Agraria y Alimentaria (INIA) Campus de Montegancedo-UPM 28223-Pozuelo de Alarcón, (Madrid), Spain
[2]CIRAD, UMR AGAP Institut, F-34398 Montpellier, France
[3]UMR AGAP Institut, Univ Montpellier, CIRAD, INRAE, Institut Agro, F-34398, Montpellier, France

Table S1: Table of simulated scenarios according to trait and environment parameters: the standard normal deviation of the environment means σ_μ, the genetic correlation between environment pairs $\rho_{j,j'}$, the constant component of the environment-specific heritability c, where $h_j^2 = c + d_j$ with d_j being a random environment-specific deviation (see Material and Methods), and the level of sparseness. When the influence of one parameter is investigated (Focus Parameter), other parameter are fixed to values of the basic scenario: $\sigma_\mu = 1$, $\rho_{j,j'} = 0.5$ for all $j \neq j'$, $h_j^2 = 0.5 + d_j$, and sparseness = 0%

Focus parameter	σ_μ	$\rho_{j,j'}$	c	Sparseness
σ_μ	0.1	0.5	0.5	0%
	1	0.5	0.5	0%
	10	0.5	0.5	0%
$\rho_{j,j'}$	1	0.2	0.5	0%
	1	0.5	0.5	0%
	1	0.8	0.5	0%
$h_j^2 = c + d_j$	1	0.5	0.2	0%
	1	0.5	0.5	0%
	1	0.5	0.8	0%
Sparseness	1	0.5	0.5	0%
	1	0.5	0.5	25%
	1	0.5	0.5	50%
	1	0.5	0.5	75%

Table S2: Selection coincidence (in percentage) between genotypes selected using true GE metric values (i.e. calculated using env-BVs) or with GE metric estimates obtained using the following estimators: No-Geno, Geno-Exp, and Geno-Exp-Var. The following selection intensities (SI) were considered: 5%, 10%, 15%, and 20%, with the corresponding number of selected genotypes indicated in parentheses. Data was simulated using the basic scenario with 0% sparseness. The values correspond to averages over 50 replicates, and the standard deviations are shown in parentheses

SI	Estimator	***Ecovalence***	***Environmental Var.***	***Lin-Binns***	***Average***
5% (10)	No-Geno	10.80% (8.29)	15.40% (12.49)	60.00% (13.25)	63.40% (16.11)
	Geno-Exp	17.40% (11.57)	31.20% (16.12)	70.40% (11.42)	69.40% (13.46)
	Geno-Exp-Var	17.80% (13.75)	30.80% (17.12)	69.40% (11.68)	-
10% (20)	No-Geno	17.10% (9.32)	26.60% (9.76)	64.30% (8.39)	67.90% (10.21)
	Geno-Exp	26.00% (8.57)	39.70% (12.99)	73.10% (8.56)	72.80% (9.27)
	Geno-Exp-Var	28.60% (12.94)	38.30% (13.65)	73.40% (8.60)	-
15% (30)	No-Geno	24.27% (7.10)	32.73% (8.27)	68.00% (9.21)	69.93% (6.62)
	Geno-Exp	36.33% (9.51)	46.67% (9.50)	73.93% (7.61)	75.67% (6.29)
	Geno-Exp-Var	38.27% (9.86)	46.93% (10.47)	73.93% (7.87)	-
20% (40)	No-Geno	31.15% (6.09)	38.85% (8.08)	72.45% (7.99)	74.55% (6.22)
	Geno-Exp	42.05% (8.29)	53.80% (8.38)	77.90% (6.67)	78.65% (5.87)
	Geno-Exp-Var	44.05% (8.58)	53.20% (9.11)	77.95% (6.97)	-

Table S3: Selection coincidence (in percentage) between genotypes selected using true GE metric values (i.e. calculated using env-BVs) or with GE metric estimates obtained using the following estimators: No-Geno, Geno-Exp, and Geno-Exp-Var. The following selection intensities (SI) were considered: 5%, 10%, 15%, and 20%, with the corresponding number of selected genotypes indicated in parentheses. Data was simulated using the basic scenario with 50% sparseness. The values correspond to averages over 50 replicates, and the standard deviations are shown in parentheses

SI	Estimator	*Ecovalence*	*Environmental Var.*	*Lin-Binns*	*Average*
5% (10)	No-Geno	5.80% (7.85)	13.20% (10.39)	42.20% (11.48)	44.40% (15.00)
	Geno-Exp	11.00% (10.55)	24.40% (14.02)	55.80% (16.30)	59.60% (13.70)
	Geno-Exp-Var	17.20% (15.39)	22.60% (15.36)	55.20% (16.44)	-
10% (20)	No-Geno	12.80% (7.96)	20.50% (10.31)	50.00% (9.69)	52.40% (9.86)
	Geno-Exp	18.60% (8.92)	33.20% (10.14)	62.90% (9.95)	66.40% (9.26)
	Geno-Exp-Var	25.70% (13.89)	31.40% (13.17)	62.80% (9.70)	-
15% (30)	No-Geno	19.67% (6.97)	26.73% (7.45)	55.47% (9.28)	59.53% (7.28)
	Geno-Exp	25.87% (7.14)	40.00% (9.38)	69.73% (8.88)	71.20% (7.31)
	Geno-Exp-Var	32.67% (12.36)	40.27% (11.95)	68.80% (10.01)	-
20% (40)	No-Geno	25.35% (7.02)	31.95% (6.39)	61.25% (7.41)	64.05% (6.56)
	Geno-Exp	31.20% (6.35)	45.15% (9.48)	72.35% (8.56)	74.40% (7.24)
	Geno-Exp-Var	38.65% (10.32)	45.65% (11.18)	71.45% (8.59)	-

Table S4: Selection coincidence (in percentage) between genotypes selected using true GE metric values (i.e. calculated using env-BVs) or with GE metric estimates obtained using the following estimators: No-Geno, Geno-Exp, and Geno-Exp-Var. The following selection intensities (SI) were considered: 5%, 10%, 15%, and 20%, with the corresponding number of selected genotypes indicated in parentheses. Data was simulated using the basic scenario with 75% sparseness. The values correspond to averages over 50 replicates, and the standard deviations are shown in parentheses

SI	Estimator	*Ecovalence*	*Environmental Var.*	*Lin-Binns*	*Average*
5% (10)	No-Geno	0.00% (0.00)	9.80% (9.79)	26.20% (12.27)	25.60% (12.80)
	Geno-Exp	7.80% (7.905)	17.20% (15.91)	43.40% (14.09)	39.60% (17.95)
	Geno-Exp-Var	13.80% (14.41)	16.40% (15.75)	39.60% (15.90)	-
10% (20)	No-Geno	8.40% (7.92)	16.30% (8.97)	36.90% (10.15)	37.70% (10.06)
	Geno-Exp	14.90% (7.79)	25.80% (11.13)	51.90% (10.25)	50.20% (11.82)
	Geno-Exp-Var	24.70% (14.65)	23.60% (11.787)	49.90% (11.45)	-
15% (30)	No-Geno	15.93% (6.26)	21.87% (7.77)	44.27% (8.03)	42.93% (7.21)
	Geno-Exp	22.60% (7.07)	33.00% (10.61)	57.33% (9.48)	56.40% (10.56)
	Geno-Exp-Var	31.80% (12.69)	31.13% (12.54)	56.80% (9.03)	-
20% (40)	No-Geno	23.00% (6.94)	25.65% (7.01)	50.20% (6.83)	49.35% (6.44)
	Geno-Exp	29.50% (8.39)	39.15% (10.13)	62.65% (7.41)	61.95% (9.44)
	Geno-Exp-Var	37.05% (9.92)	37.00% (11.02)	61.80% (8.10)	-

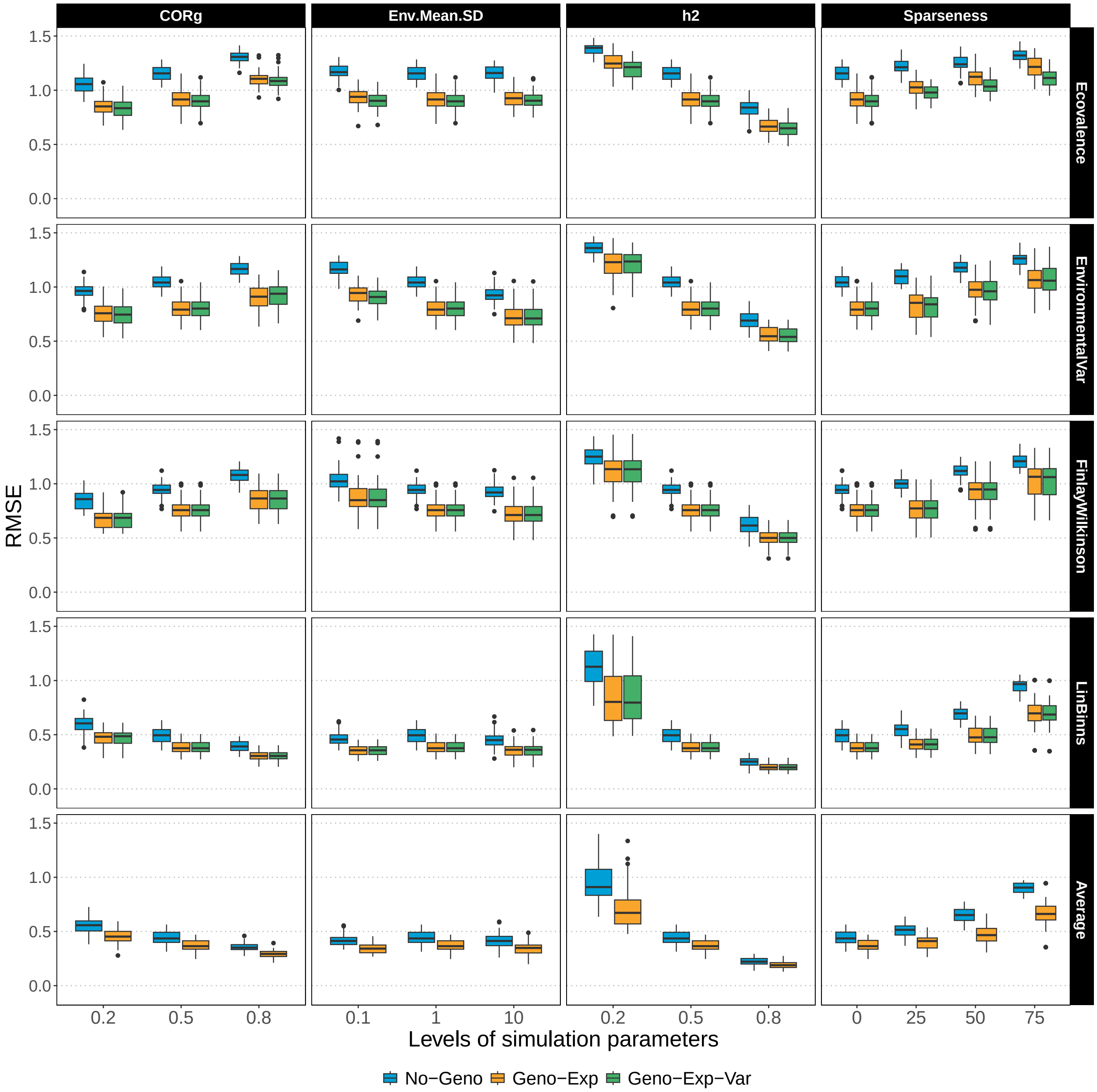

Figure S1: Scaled root mean squared error (RMSE, y-axis) between the GE metrics calculated from simulated environment-specific breeding values and the estimates obtained from No-Geno (blue), Geno-Exp (orange), and Geno-Exp-Var (green) estimators for all simulated parameter levels (x-axis): standard deviation of the environmental means (σ_μ = Env.Mean.SD), genetic correlation between environments ($\rho_{j,j'}$ = CORg), heritability (h_j^2 = h2), and sparseness of the data (0%, 25%, 50%, and 75%). All simulation parameters were modulated one by one around a basic scenario

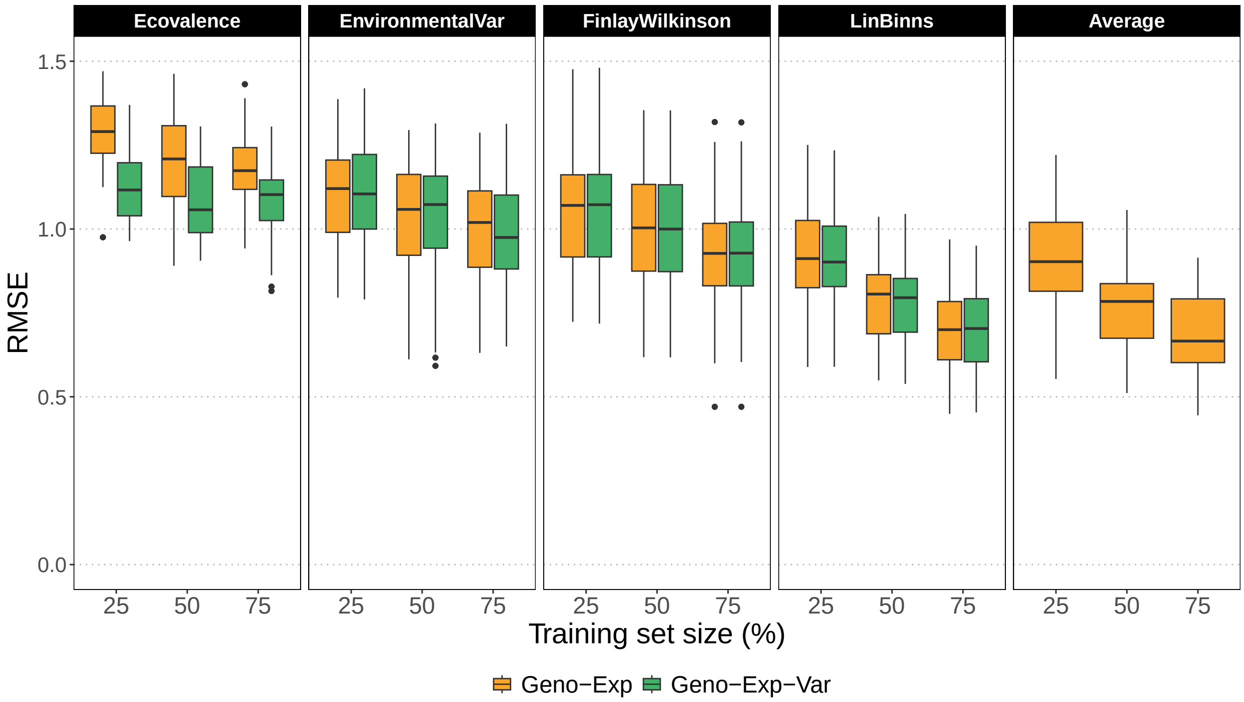

Figure S2: Scaled root mean squared error (RMSE, y-axis) of each GE metric considering the basic simulated scenario. The RMSE was calculated by comparing GE metrics predictions and true values (i.e. calculated using simulated env-BVs), and was assessed by cross-validation considering different training set sizes (x-axis) in percentage of the total number of genotypes (25%, 50%, and 75%). Two estimators were compared: Geno-Exp (orange) and Geno-Exp-Var (green)

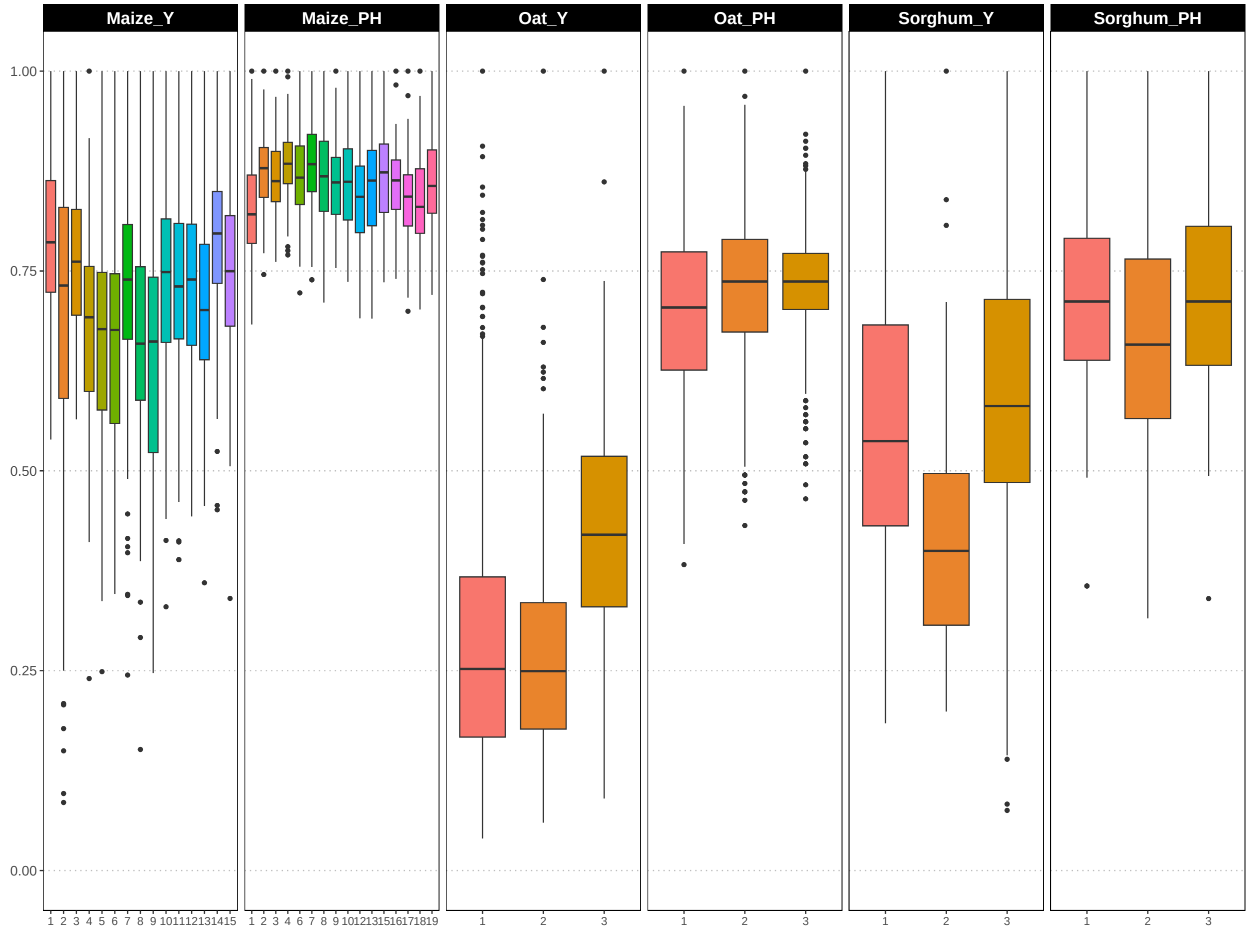

Figure S3: Boxplots of standardized adjusted means for yield (Y) and plant height (PH) according to the environment for each empirical datasets: Maize, Oat, and Sorghum

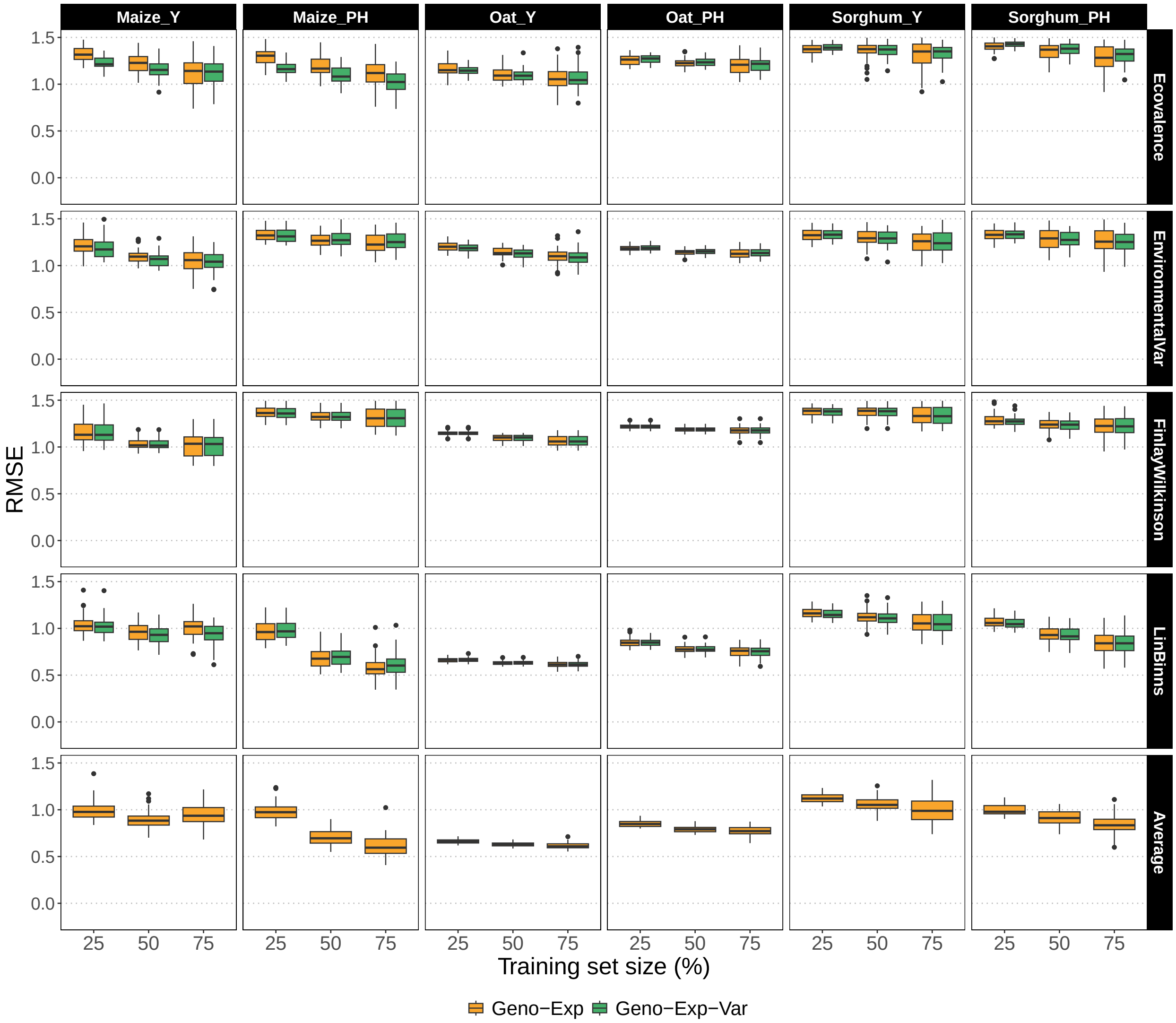

Figure S4: Scaled root-mean-squared error (RMSE, y-axis) of each GE metric for empirical datasets (Maize, Oat, and Sorghum) evaluated for yield (Y) and plant height (PH). The RMSE was calculated by comparing GE metrics predictions and reference values (i.e. calculated using adjusted means), and was assessed by cross-validation considering different training set sizes (x-axis) in percentage of the total number of genotypes (25%, 50%, and 75%). Two estimators were compared: Geno-Exp (orange) and Geno-Exp-Var (green)
